# Genome wide survey, evolution and expression analysis of PHD finger genes reveal their diverse roles during the development and abiotic stress responses in *Brassica rapa* L.

**DOI:** 10.1186/s12864-019-6080-8

**Published:** 2019-10-24

**Authors:** Intikhab Alam, Cui-Cui Liu, Hong-Liu Ge, Khadija Batool, Yan-Qing Yang, Yun-Hai Lu

**Affiliations:** 1 Key Laboratory of Ministry of Education for Genetics, Breeding and Multiple Utilization of Crops, College of Crop Science, Fujian Agriculture and Forestry University, Fuzhou, 350002 China; 2 Marine and Agricultural Biotechnology Laboratory, Institute of Oceanography, Minjiang University, Fuzhou, 350108 China

**Keywords:** *Brassica rapa*, PHD finger genes, Gene duplication, Evolution, Genes expression, Abiotic stress

## Abstract

**Background:**

Plant homeodomain (PHD) finger proteins are widely present in all eukaryotes and play important roles in chromatin remodeling and transcriptional regulation. The PHD finger can specifically bind a number of histone modifications as an “epigenome reader”, and mediate the activation or repression of underlying genes. Many PHD finger genes have been characterized in animals, but only few studies were conducted on plant PHD finger genes to this day. *Brassica rapa* (AA, 2n = 20) is an economically important vegetal, oilseed and fodder crop, and also a good model crop for functional and evolutionary studies of important gene families among *Brassica* species due to its close relationship to *Arabidopsis thaliana.*

**Results:**

We identified a total of 145 putative PHD finger proteins containing 233 PHD domains from the current version of *B. rapa* genome database. Gene ontology analysis showed that 67.7% of them were predicted to be located in nucleus, and 91.3% were predicted to be involved in protein binding activity. Phylogenetic, gene structure, and additional domain analyses clustered them into different groups and subgroups, reflecting their diverse functional roles during plant growth and development. Chromosomal location analysis showed that they were unevenly distributed on the 10 *B. rapa* chromosomes. Expression analysis from RNA-Seq data showed that 55.7% of them were constitutively expressed in all the tested tissues or organs with relatively higher expression levels reflecting their important housekeeping roles in plant growth and development, while several other members were identified as preferentially expressed in specific tissues or organs. Expression analysis of a subset of 18 *B. rapa* PHD finger genes under drought and salt stresses showed that all these tested members were responsive to the two abiotic stress treatments.

**Conclusions:**

Our results reveal that the PHD finger genes play diverse roles in plant growth and development, and can serve as a source of candidate genes for genetic engineering and improvement of *Brassica* crops against abiotic stresses. This study provides valuable information and lays the foundation for further functional determination of PHD finger genes across the *Brassica* species.

## Background

Zinc finger proteins are abundantly present in both prokaryotic and eukaryotic genomes, including the plant kingdom [1–8]. They are characterized by the presence of one or more sequence motifs in which cysteines and/or histidines coordinate one or more zinc atoms to form stable local peptide structures (zinc fingers, ZFs) that are required for their specific functions [2, 4]. The zinc finger was first identified in *Xenopus laevis* transcription factor IIIA (TFIIIA) in 1985 [9], and the three dimensional solution structure of a single zinc finger was first reported in 1989 [10]. Since then, various other zinc binding motifs have been identified and characterized, and as high as 30 types of Zinc finger proteins were currently identified in human genome based on the zinc-finger domain structure [11, 12]. The most common types of zinc finger proteins include C2H2, RING (*really interesting new gene*), PHD (*plant homeodomain*), and LIM (*Lin-ll, Isl-1 and Mec-3*) families [2, 12, 13]. These varied zinc finger domains enable different proteins to interact specifically with cognate DNA, RNA, proteins, lipids (or membrane), and small molecules through hydrogen bonds and hydrophobic interactions [14–16]. Proteins containing zinc finger domain (s) were found to play important roles in various molecular, physiological and cellular processes in cells or tissues, and some of them may function as part of a large regulatory network that senses and responds to different environmental stimuli, and regulate different signal transduction pathways and controlling processes, such as development and programmed cell death [2–8, 12, 17–21].

The PHD finger was first identified in *Arabidopsis thaliana* transcription factor HAT3.1 (a homeodomain-containing protein) and its maize homolog Zmhox1a in 1993 [22]. Since then, many other PHD-finger proteins have been identified in various eukaryotes, including the yeast [23, 24], Drosophila [25, 26] and human [12, 27, 28]. The PHD finger can be defined as a Cys-rich domain of approximately 50~80 amino acids with spatially conserved 8 metal ligands arranged as unique Cys4-His-Cys3 pattern in 4 pairs which can chelate two Zn^2+^ atoms and form a cross-brace structure [13, 29, 30]. The PHD finger can specifically bind a number of histone modifications as an “epigenome reader”, and mediate the activation or repression of underlying genes [30–36]. In human, mutations in PHD fingers or deletions of these domains are linked to a number of diseases such as cancer, mental retardation, and immunodeficiency [32, 33]. In plant, the PHD domains were found to be involved in the transcriptional regulation of developmental processes such as meiosis and postmeiotic events during pollen maturation, embryo meristem initiation and root development, germination, flowering time, etc. [36].

The Brassicaceae or Cruciferae is one of the most important families of flowering plants, containing some 338 genera and approximately 3709 species, with an extreme high level of morphological diversity [37, 38]. The family includes a number of economically important species of the genus *Brassica* cultivated worldwide as vegetables, oil seed crops, condiments and fodder crops, as well as the extensively studied model plant *Arabidopsis thaliana* [39]. The genomic relationships among the six cultivated *Brassica* species, including *B. rapa* (2n = 20, AA genome, 529 Mb genome size), *B. nigra* (2n = 16, BB, 632 Mb), *B. oleracea* (2n = 18, CC, 696 Mb), *B. juncea* (2n = 36, AABB, 1068 Mb), *B. napus* (2n = 38, AACC, 1132 Mb) and *B. carinata* (2n = 34, BBCC, 1284 Mb), has long been established as the Triangle of U [40, 41]. Previous studies revealed that all the species of the tribe Brassiceae shared a common whole-genome triplication (WGT) event occurred ~ 15.9 million years ago (MYA) just after the divergence of their ancestor from that of *A. thaliana* (tribe Arabideae) [42–45]*.* This whole genome triplication event was followed by genome diploidization involving substantial genome reshuffling and gene losses in duplicated genomic blocks, and resulted in three subgenomes with different degree of gene losing, e.g. least fractionized (LF), moderately fractionized (MF1) and most fractionized (MF2) subgenomes [46, 47]. *B. rapa* is an important, worldwide cultivated crop with various morphotypes, such as leafy vegetables, turnips and oilseed rape [38, 48]. Because of its smallest genome size of the genus *Brassica*, rapid life cycle, high morphological diversity, and origin from a common hexaploid ancestor as all other members of the tribe Brassiceae, *B. rapa* became a model plant for genetic, genomic and evolutionary studies in *Brassica* species [47, 49]. The complete sequencing of the *B. rapa* genome makes it possible to analyze some important gene families at a whole genome level [49–56].

Abiotic stresses, especially salt and drought stresses, affect many aspects of plant physiology and metabolism, and cause severe crop yield losses around the world [57]. *Brassica* crops are mainly grown in arid and semiarid areas, and they are the most affected by drought and salinity among the major food crops [58]. Several drought or salt-tolerant genes isolated in *A. thaliana* as well as in *Brassica* crops showed great potential for genetic improvement of plant tolerance [58]. In several previous studies [59–67], some PHD finger genes were found to be highly responsive to abiotic stresses, including salt and drought stresses, suggesting that they might play important roles for the response and adaptation to abiotic stresses in plants. In the current paper, we reported the identification and comprehensive analysis of the PHD finger genes in *B. rapa* genome. Our results lay a foundation for further functional characterization of PHD finger genes among *Brassica* species.

## Results

### Identification and characterization of PHD finger genes in *B. rapa*

A total of 145 non-redundant predicted PHD finger proteins containing 233 PHD finger domains were identified from the Brassica Database (BRAD), of which 92 (65.7%), 27 (18.6%), 17 (11.7%) and 9 (6.2%) contain one, two, three and four putative PHD domain (s), respectively (Additional file 2: Table S1). The details of these *B. rapa* PHD protein genes, such as locus name, chromosome location, CDS and amino acid lengths, protein masse, and isoelectric points (pIs), were summarized in Additional file 2: Table S1. We also identified 16 PHD-suspected domain-containing proteins that contain each an imperfect PHD motif (Additional file 2: Table S2).

The identified 233 *B. rapa* PHD domains were extracted from their corresponding protein sequences, and archived in Additional file 2: Table S1. Based on these domain sequences, a multiple sequence alignment, a sequence logo of the over-represented residues and a phylogenetic tree were generated and illustrated in Additional file 1: Figure S1, S2 and S3, respectively. Additional file 1: Figures S1 and S2 illustrated the conservation of eight metal ligands as well as the spacing between them, while Additional file 1: Figure S3 showed the close evolutionary inter-relationships among some of these PHD domains and their multiplication during the *B. rapa* genome evolution.

**Fig. 1:**
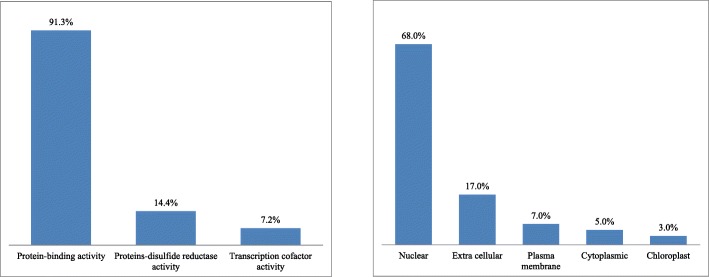
Distribution of GO molecular function terms (**a**) and sub-cellular localization (**b**) of 145 *Brassica rapa* PHD finger proteins

In order to gain a global idea about the function of the *B. rapa* PHD finger genes, we retrieved their associated Gene Ontology (GO) terms from Phytozome database, and performed the prediction of subcellular localization by CELLO software (Additional file 2: Table S1). The GO Molecular Function term is available for 138 out of 145 (95%) identified PHD finger proteins, which can be classified into protein-binding activity (126/138 = 91.3%), proteins-disulfide reductase activity (20/138 = 14.4%), and transcription cofactor activity (10/138 = 7.2%) (Fig. 1a). Ninety-eight out of 145 (67.6%) *B. rapa* PHD finger proteins were predicted to be localized in nucleus, 25 (17.2%) in extracellular, 10 (6.9%) in plasma membrane, 7 (4.8%) in cytoplasm, and 5 (3.5%) in chloroplast (Fig. 1b). The distribution of 145 *B. rapa* PHD proteins containing one to four PHD domain (s) in different cellular compartments was summarized in Table 1. We observed that near 80% of 1-PHD domain-containing *B. rapa* PHD finger proteins were localized in nucleus, while only 12.0% in extracellular, 7.6% in cytoplasm, 2.2% in chloroplast, and 0% in plasma membrane. Among both the 2- and 3-PHD domain-containing proteins, a little more than 50% were localized in nucleus, about 20–30% in extracellular, about 20% in plasma membrane, 11 or 0% in chloroplast, and 0% in cytoplasm. Among the nine 4-PHD domain-containing proteins, three (33%) were localized in nucleus, four (44%) in extracellular, and two (22%) in plasma membrane.

**Table 1:** Distribution of 145 *Brassica rapa* PHD proteins containing one to four PHD domain (s) in different cellular compartments predicted by using the CELLO v2.5 software (http://cello.life.nctu.edu.tw). Values in parentheses indicate the percentages of proteins localized in a given cellular compartment per total 1-, 2-, 3- or 4-PHD domain-containing proteins

No. of PHD domain	No. of proteins	Nuclear	Cytoplasm	Extracellular	Plasma membrane	Chloroplast
1	92	72 (78.3)	7 (7.6)	11 (12.0)	0 (0.0)	2 (2.2)
2	27	14 (51.9)	0 (0.0)	5 (18.5)	5 (18.5)	3 (11.1)
3	17	9 (52.9)	0 (0.0)	5 (29.4)	3 (17.6)	0 (0.0)
4	9	3 (33.3)	0 (0.0)	4 (44.4)	2 (22.2)	0 (0.0)
Total No.	145	98	7	25	10	5

### Phylogenetic and gene structure analyses of *B. rapa* PHD finger proteins

**Fig. 2:**
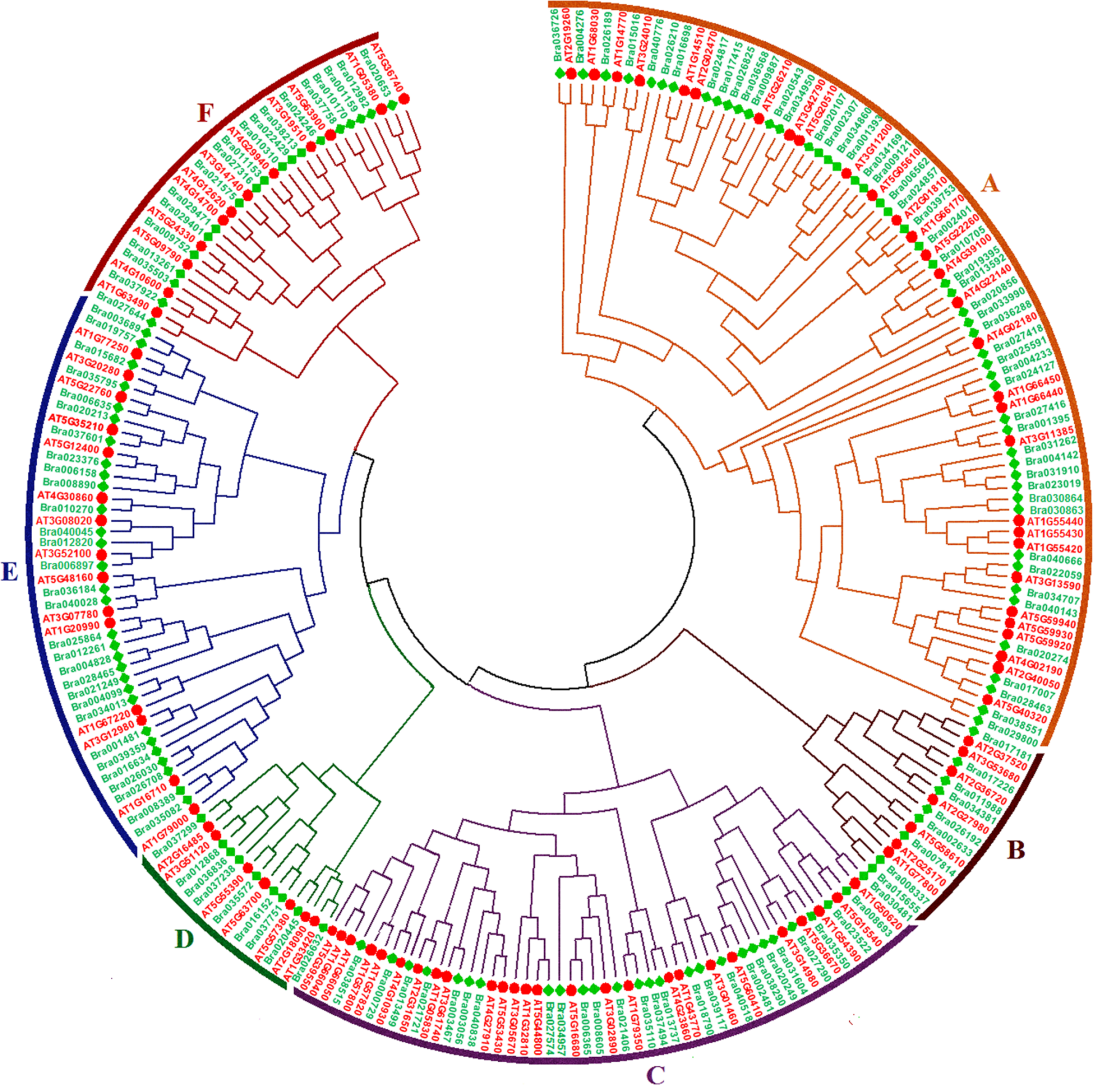
Phylogenetic tree analysis of the PHD finger proteins from *Arabidopsis* and *Brassica rapa*. The tree was determined by using MEGA6.06 software with the Neighbor–Joining (NJ) algorithm and a bootstrap analysis of 1000 replicates. The PHD finger proteins were clustered into six major groups (A-F). Proteins from *Arabidopsis* and *B. rapa* are indicated by red and green colors, respectively

To gain insights into the evolutionary relationships among *B. rapa* PHD finger proteins, a phylogenetic tree was generated based on the sequences of 145 *B. rapa* as well as 97 *A. thaliana* PHD finger proteins (Fig. 2). The result showed that these PHD finger proteins could be divided into six major groups, named A, B, C, D, E and F, within which the orthologous or homologous proteins from *B. rapa* and *A. thaliana* were closely clustered together (Fig. 2). The largest group A contains 51 *B. rapa* and 30 *A. thaliana* PHD finger genes. The group B contains 9 *B. rapa* and 7 *A. thaliana* PHD finger genes. The group C contains 27 *B. rapa* and 26 *A. thaliana* PHD finger genes. The group D contains 9 *B. rapa* and 7 *A. thaliana* PHD finger genes. The group E contains 30 *B. rapa* and 15 *A. thaliana* PHD finger genes. The group F contains 19 *B. rapa* and 12 *A. thaliana* PHD finger genes. Obviously, groups A, C, E and F can be further divided into several subgroups. An enlarged phylogenetic tree including PHD finger proteins from *A. thaliana*, *B. rapa*, *Oryza sativa*, *Populus trichocarpa* and *Zea mays* was also generated and presented in Additional file 1: Figure S4, showing the orthologous relationships and high evolutionary conservation between PHD finger proteins of different species. The Gene Ontology terms associated with these 97 *A. thaliana* PHD finger genes were summarized in Additional file 2: Table S3, showing the rich information concerning their functions in *A. thaliana* that can be used to explore the functions of their corresponding orthologs in *Brassica* crops.

**Fig. 3:**
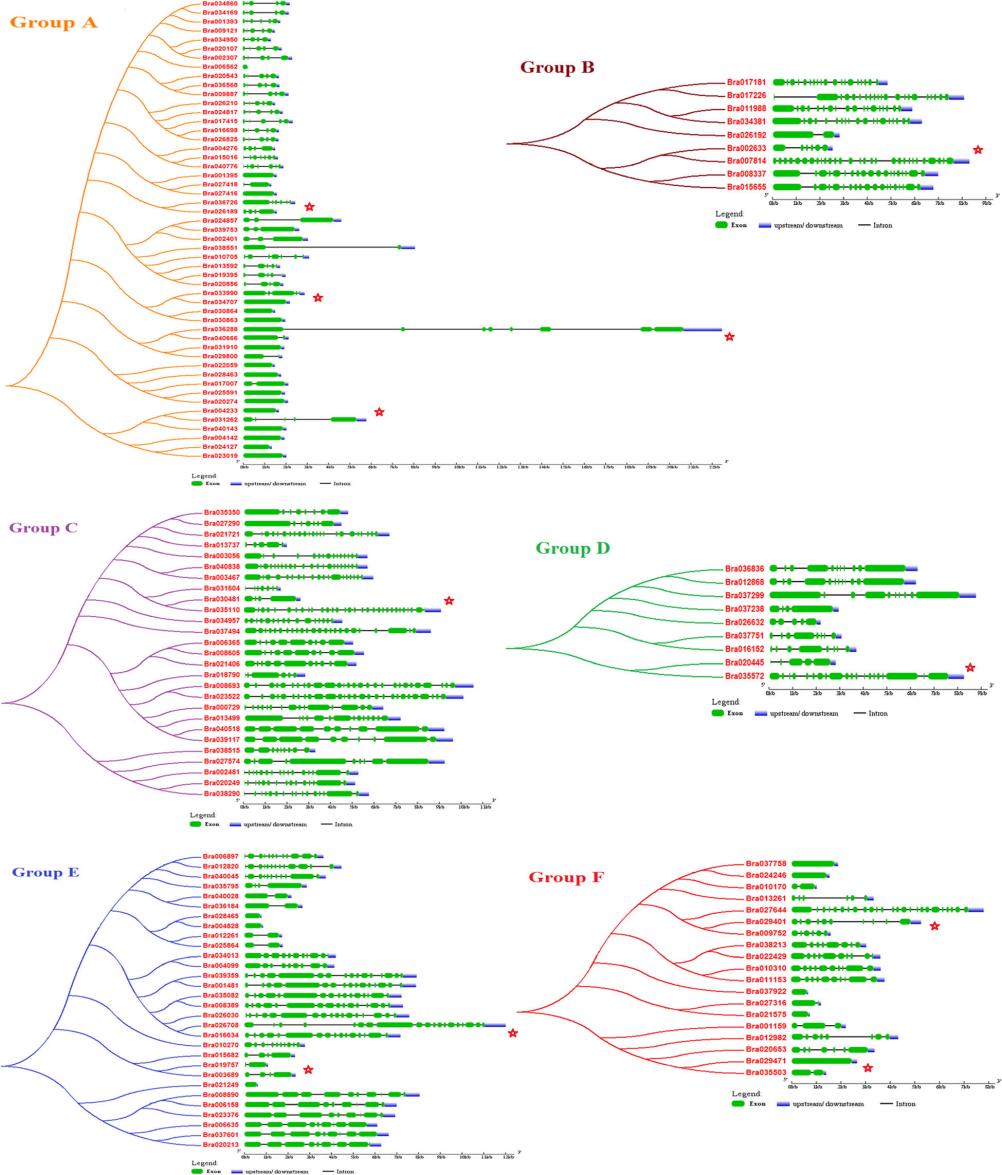
Phylogenetic relationships and gene structure of PHD finger genes in *Brassica rapa*. The tree was generated by using Neighbor-Joining method with 1000 bootstrap replicates. The tree shows six major phylogenetic groups (group A to F) indicated with six differently colored backgrounds. Green color boxes represent exons and grey color lines indicate introns, and the untranslated regions (UTRs) are represented by blue color boxes. The sizes of exons and introns can be estimated using the scale at the bottom. The tightly clustered genes with remarkable differences in their gene structures are indicated by red stars

Intron loss or gain is another important evolutionary mechanism that generates gene structural diversity and complexity, and contributes to the functional diversity and divergence during the evolution of multi-gene families in plants [68, 69]. To obtain insights into the structural variation of *B. rapa* PHD-finger protein genes, we analyzed their exon/intron organization from the genomic sequences of individual *B. rapa* PHD-finger protein genes, in relation to their phylogenic tree by groups extracted from the Fig. 2 (Fig. 3). The result showed that the most closely related members tended to be clustered together and shared similar exon/intron structures but with exceptions, such as between Bra036726 and Bra026189 in group A, between Bra02633 and Bra07814 in group B, between Bra030481 and Bra035110 in group C, between Bra020445 and Bra035572 in group D, between Bra026708 and Bra016634 in group E, and between Bra029401 and Bra009752 in group F. Among the identified 145 *B. rapa* PHD finger proteins, 26 (17.9%) (including 19 in group A, one in group C, four in group E, and six in group F) possess each 0 intron, 15 (10.3%) (five in group A, one in group B, one in group C, five in group E, three in group F) contain each 1 intron, while the remaining 104 contain each 2–30 introns (Bra035110 in group C contains 30 introns, Bra007814 in group B contains 28 introns). Bra036288 in group A is the longest gene covering a genomic sequence as long as ~ 21 kb, contrasting to the shortest member Bra006562 covering a genomic sequence of ~ 0.2 kb (Fig. 3).

Previous studies have shown that tandem PHD fingers can fold as one functionally cooperative unit and be used to read more complex combinations of histone modifications, thus reinforcing the notion that the unequal numbers of PHD-finger domains detected in each protein sequence may contribute to their functional diversity and complexity [35, 70]. The distribution of 145 *B. rapa* PHD proteins containing one to four PHD domain (s) in different phylogenetic groups of Fig. 2 was summarized in Table 2. We observed that the proportion of 1-PHD domain-containing proteins was very high in group D (88.9%) and C (85.2%), followed by F (63.2%), E (60.0%), A (54.9%) and B (33.3%). The twenty-seven 2-PHD domain-containing proteins were distributed into group A (15.7%), B (44.4%), C (14.8%), E (13.3%) and F (36.8%), while the 17 3-PHD domain-containing proteins into group A (16.7%), D (11.1%) and E (23.3%), and the nine 4-PHD domain-containing proteins into A (11.7%), B (22.2%) and E (3.3%).

**Table 2:** Distribution of 145 *Brassica rapa* PHD proteins containing one to four PHD domain (s) in the different phylogenetic groups of Fig. 2. Values in parentheses indicate the percentages of 1-, 2-, 3- or 4-PHD domain-containing proteins per total proteins of each phylogenetic group

Phylogenetic group	No. of genes	1-PHD domain-containing	2-PHD domain-containing	3-PHD domain-containing	4-PHD domain-containing
A	51	28 (54.9)	8 (15.7)	9 (16.7)	6 (11.7)
B	9	3 (33.3)	4 (44.4)	0 (0.0)	2 (22.2)
C	27	23 (85.2)	4 (14.8)	0 (0.0)	0 (0.0)
D	9	8 (88.9)	0 (0.0)	1 (11.1)	0 (0.0)
E	30	18 (60.0)	4 (13.3)	7 (23.3)	1 (3.3)
F	19	12 (63.2)	7 (36.8)	0 (0.0)	0 (0.0)
Total No.	145	92	27	17	9

### Additional domain analysis outside of PHD finger domain

For each of the identified 145 PHD finger protein, the presence or not of any additional known domain outside of the PHD finger domain (s) was inspected through the Smart analysis. A total of 56 additional known domains were identified, allowing classifying the 145 PHD finger proteins into 28 groups and subgroups (Additional file 2: Table S4). The largest group (group 1) includes 42 members (42/145 = 29.0%) which all contain no other additional domain besides the 1–4 PHD domain (s). The second group includes 15 members (15/145 = 10.3%) which contain each a DUF3594 domain besides a single PHD domain. The third groups includes 9 members (9/145 = 6.2%) which contain each a KAT11, a ZnF_ZZ and a ZnF_TAZ domain besides a single PHD domain. The other 25 groups include each 1–8 members with 1–5 additional known domains. These additional known domains may be involved in protein-protein interaction (Ald_Xan_dh_C2, Coiled-coil, JmJC, PWWP), protein binding (GYF, SWIB), histone binding/acetylation/methylation (BAH, Cohisin Heat, DUF295, DUF1086, DUF3594, DUF1087, ING, KAT11, NIPPED-B_C, Post-SET, SET, SRA), nucleic acid binding (AAA, Ald_Xan_Dh_C2, ARID, AT hook, DDT, DEXDc, DNMT1, HELICc, Helicase_C_4, MBD, PLUS3, PPR, Res III, SANT, SAP, WHIIM, Znf-C2H2, Znf-C5HC2), and Zinc ion binding (Znf-C2H2, Znf-C5HC2, Znf-CCCH, Znf-TAZ, Znf-UBA, Znf-ZZ). Other known domains such as AMP-Binding (catalytic activity), C1 (intracellular signal transduction), DYW_deaminase, ELM2, EloA-BP1, FLU-1, FYRC, FYRN, JAS (jasmonate signaling), MBOAT_2, Oberon_cc and Transmembrane, were also detected.

Table 3 summarized the distribution of 56 additional known domains in the different phylogenetic groups of Fig. 2. We observed that 46 out of 56 (82.1%) additional domains were specific to a single group, i. e., 7 were specific to group A, 3 to group B, 19 to group C, 6 to group D, 5 to group E, and 6 to F; five out of 56 (8.9%) were simultaneously present in two groups; four out of 56 (7.1%) were simultaneously present in three groups; and one (Coiled-coil domain) out of 56 (1.8%) was simultaneously present in five (A, B, C, E and F) of six groups. The group C contains as high as 26 types of additional domains, compared to a value of 11, 7, 8, 11 and 10 for group A, B, D, E and F, respectively. It is worth to remark that, among the 51 members of group A, 15 (29.4%) shared DUF3594 domain known for involving in histone binding and regulation of transcription activities, and eight (15.7%) shared the C1 domain known for intracellular signal transduction activity. Among the nine members of group B, five (55.6%) shared JAS domain known for jasmonate signaling activity. Group C (27 members) contains very diverse additional domains, including Coiled-coil (14.8%) and PWWP (14.8%) known for protein-protein interaction activity, Post-SET (14.8%) and SET (14.8%) for histone methyltransferase activity, and SAP (11.1%) for DNA-binding involved in chromosomal organization. Group D (9 members) contains additional domains such as GYF (44.4%) and SWIB (66.7%) known for protein binding activity, and PLUS3 (44.4%) for DNA binding activity. Group E (30 members) contains additional domains such as DDT (20.0%) known for DNA binding activity, KAT11 (30.0%) for histone acetylation activity, and Znf-TAZ (30.0%) and Znf-ZZ (30.0%) for zinc ion binding activity. Group F (19 members) contains additional domains such as HOX (21.1%) and Znf-C5HC2 (5.3%) known for DNA binding activity, and JmjC (5.3%) for demethylase activity.

**Table 3:** Distribution of 56 additional known domains besides the PHD finger domains in the different phylogenetic groups of Fig. 2. Values in parentheses indicate the percentages of given additional domain-containing proteins per total proteins of each phylogenetic group

Additional domain	Group A (59 proteins)	Group B (9 proteins)	Group C (27 proteins)	Group D (9 proteins)	Group E (30 proteins)	Group F (19 proteins)
AAA						1 (5.3)
Ald_Xan_dh_C2	1 (2.0)					
AMP-binding	1 (2.0)					
ARID			1 (3.7)			
AT hook					2 (6.7)	
BAH	4 (7.8)					
BRCT			1 (3.7)			
C1	8 (15.7)				3 (10.0)	
CHROMO		1 (11.1)				
Cohesin Heat			2 (7.4)			
Coiled-coil	2 (3.9)	1 (11.1)	4 (14.8)		3 (10.0)	2 (10.5)
DDT					6 (20.0)	
DEXDc			1 (3.7)			
DNMT1-RFD				1 (11.1)		
DUF1086		1 (11.1)				
DUF1087		1 (11.1)	1 (3.7)			
DUF295						1 (5.3)
DUF3594	15 (29.4)					
DYW_deaminase			1 (3.7)			
ELM2	1 (2.0)					
EloA-BP1			1 (3.7)			
FLU-1						1 (5.3)
FYRC			1 (3.7)			
FYRN			1 (3.7)			
GYF				4 (44.4)		
Helicase_C_4			1 (3.7)			
HELICc		1 (11.1)				
HOX						4 (21.1)
ING	2 (3.9)					
JAS		5 (55.6)	2 (7.4)			1 (5.3)
JmjC						1 (5.3)
KAT11					9 (30.0)	
MBD			1 (3.7)			
MBOAT_2				1 (11.1)		
Nipped-B_C			2 (7.4)			
Oberon-cc		1 (11.1)			2 (6.7)	
PLUS3				4 (44.4)		
Post-SET			4 (14.8)		1 (3.3)	
PPR			1 (3.7)			
PWWP			4 (14.8)			
Res III			1 (3.7)			
RING	4 (7.8)		2 (7.4)			1 (5.3)
SANT			1 (3.7)			
SAP			3 (11.1)			
SET			4 (14.8)		1 (3.3)	2 (10.5)
SRA			1 (3.7)			
SWIB				6 (66.7)		
Transmembrane	1 (2.0)			1 (11.1)		
WHIM1			2 (7.4)	1 (11.1)	2 (6.7)	
Znf-C2H2	1 (2.0)					
Znf-C5HC2						1 (5.3)
Znf-CCCH				1 (11.1)		
Znf-MIZ			3 (11.1)			
Znf-TAZ					9 (30.0)	
Znf-UBR			1 (3.7)			
Znf-ZZ					9 (30.0)	

### Chromosomal distribution, gene duplication and syntenic relationships

**Fig. 4:**
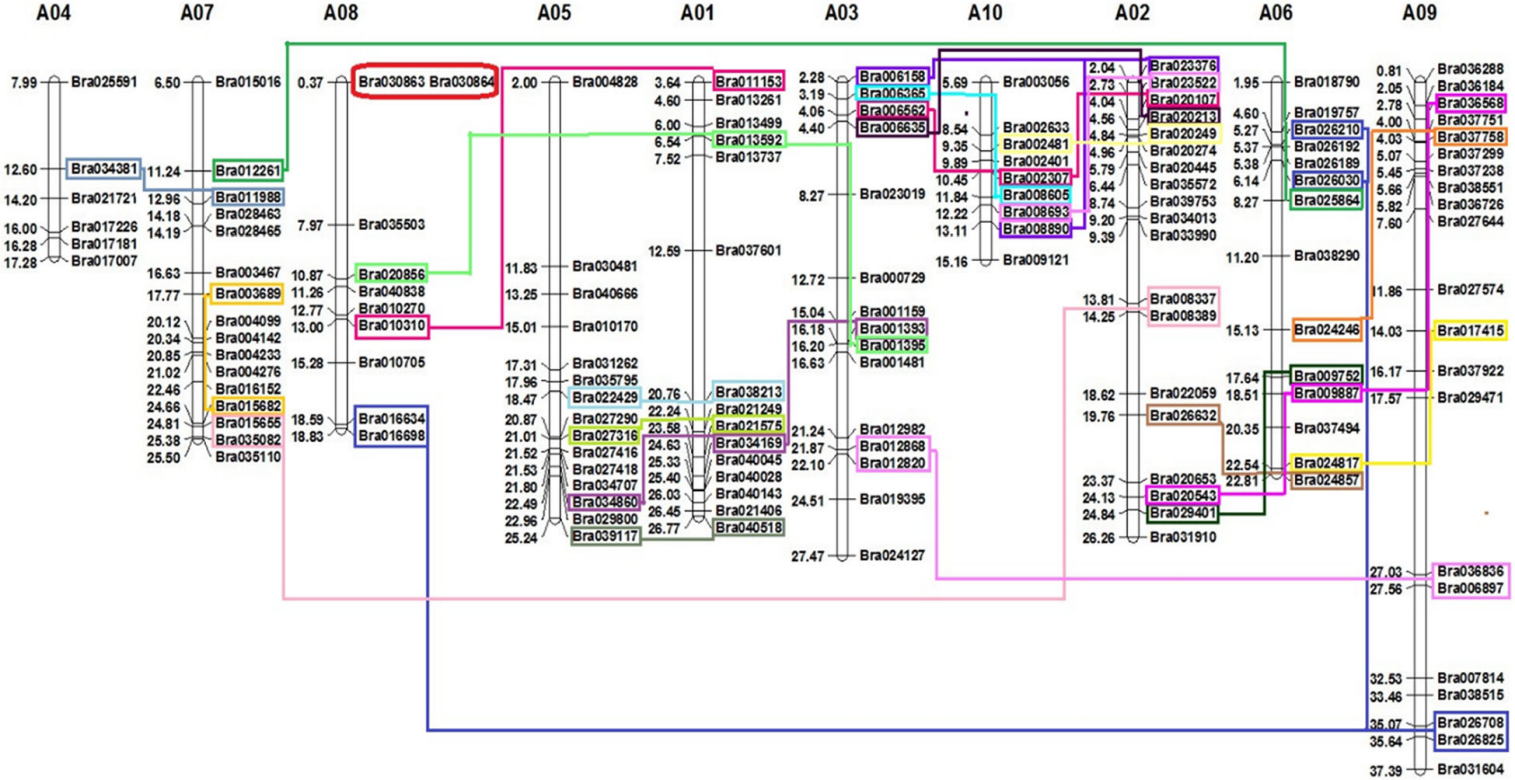
Distribution of 140 PHD finger genes on 10 chromosomes of *Brassica rapa*. The 140 *BrPHD* genes unevenly located on each conserved collinear blocks of the chromosomes. Chromosome number (A01-A10) is indicated at the *top* of each chromosome. Gene name is indicated on the *right side* of each chromosome. The physical position (Mb) of each mapped gene is indicated on the left side of each chromosome. The genes located on duplicated chromosomal segments are framed by same colors and connected by *same color lines* between the two relevant chromosomes. The tandem repeated genes are marked by red color on the chromosomes

Based on the chromosome location data of each identified PHD finger gene retrieved from BRAD database (Additional file 2: Table S1), 140 out of 145 (96.6%) *B. rapa* PHD finger genes were mapped into the 10 chromosomes of *B. rapa* (Fig. 4)*,* while the remaining 5 PHD genes were not mapped to a specific chromosome because they were currently assigned to isolated scaffolds. Our results showed that these PHD finger genes were unevenly distributed across the 10 *B. rapa* chromosomes. The number of mapped PHD finger genes is 21 on A09, 19 on A02, 16 on A07, 15 on A01, 15 on A03, 15 on A05, 14 on A06, 10 on A08, 9 on A10, and 6 on A04. *B. rapa* PHD finger genes tend to be clustered in some chromosomal regions. Our mapping analysis showed also that 58 out of 140 (41.4%) *B. rapa* PHD finger genes were involved in segmental duplication and only two genes (1.4%) were involved in tandem duplication (Fig. 4).

*Brassica* species have all undergone a whole genome triplication (WGT) event ~ 15.9 MYA following their divergence from the *Arabidopsis* lineage ~ 20 MYA [42, 47, 71–73]. *B. rapa* is considered as a paleohexaploid, and contains three subgenomes commonly called as least fractionized (LF), moderately fractionized (MF1) and most fractionized (MF2) [47, 71–73]. The syntenic relationships between the PHD finger genes of *B. rapa* and *A. thaliana* were determined from BRAD database, and summarized in Additional file 2: Table S5. Among the 145 *B. rapa* PHD finger genes, 59 (40.7%) were assigned on LF, 46 (31.7%) on MF1, and 40 (27.6%) on MF2. In seven cases, the three paralogous copies were simultaneously conserved on the three subgenomes LF, MF1 and MF2, while in 21 cases, only two of the three expected paralogous copies were conserved, and in 68 cases, only one of the three expected paralogous copies was conserved. One hundred eighteen out of 145 (81.4%) *B. rapa* PHD finger genes had their syntenic orthologs *in A. thaliana,* covering 23 blocks of seven chromosomes of ancestral translocation Proto-Calepineae Karyotype (tPCK) [43, 44, 71–73]. Twenty seven out of 145 (18.6%) *B. rapa* PHD finger genes didn’t have their syntenic orthologs *in A. thaliana,* while 18 out of 97 (18.6%) *A. thaliana* PHD finger genes didn’t have their syntenic orthologs *in B. rapa* (Additional file 2: Table S5).

### Expression analysis of *B. rapa* PHD finger genes in different tissues

**Fig. 5:**
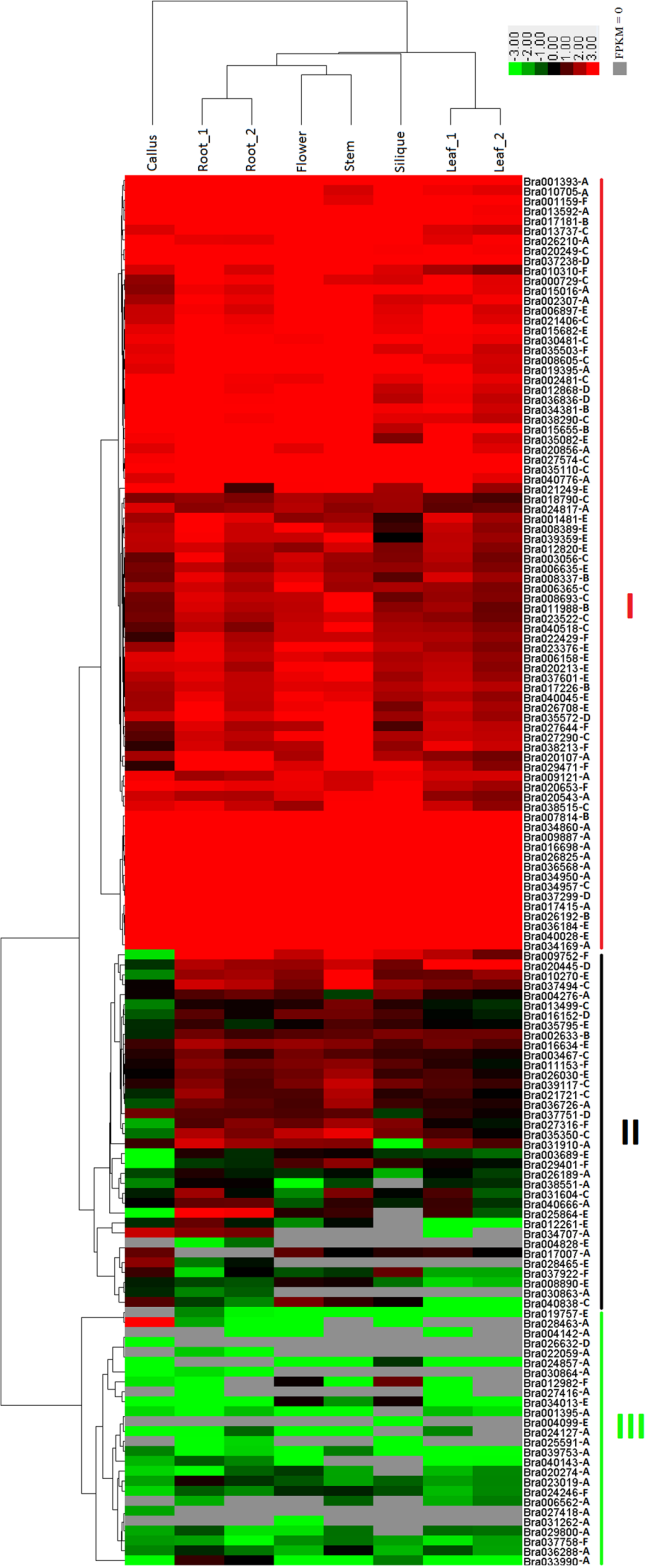
Expression profile of 140 Brassica rapa PHD finger genes in different tissues revealed by clustering analysis of RNA-Seq data. The 140 genes were divided into three major groups (I-III) based on the log2-transformed fragments per kilobase of transcript per million fragments mapped (FPKM) values. The scale representing the relative signal values is shown above. The tissue types are indicated on the top. The individual gene names are indicated on the right side

The expression patterns of individual *B. rapa* PHD finger genes in different tissues (callus, root, stem, leaf, flower and silique) were analyzed based on a publicly available *B. rapa* RNA-Seq transcriptomic dataset [74]. Except for Bra002401, Bra004233, Bra010170 and Bra021575, the expression data of 141 other *B. rapa* PHD finger genes were available from the dataset, of which one (Bra013261) showed an expression value of zero for all the six tissues, while the remaining 140 genes were expressed in at least one of the six tissues., A clustered heat map displaying the expression patterns of the 140 *B. rapa* PHD finger genes in callus, root, stem, leaf, flower and silique was generated based on their log2-transformed fragments per kilobase of transcript per million fragments mapped (FPKM) values (Fig. 5). The result showed that these 140 *B. rapa* PHD finger genes were clustered into three major groups with subgroups. The group I (biggest) includes 78 genes, which were almost all constitutively expressed in all the tested tissues with relatively higher expression levels. The group II includes 36 genes, preferentially (> 2-folds higher) expressed in one or more tissues with relatively higher expression levels. For example, Bra028465 (corresponding to *Arabidopsis* gene At5g40590, a cysteine/histidine-rich C1 domain family protein gene) was preferentially expressed in root, but very lowly (or not) expressed in other tested tissues; Bra025864 (corresponding to At1g20990, another cysteine/histidine-rich C1 domain family protein gene) was preferentially expressed in root, and only very lowly expressed in other tissues; Bra029401 (corresponding to At5g24330 or *Arabidopsis* TRITHORAX-RELATED protein 6, ATXR6) was preferentially expressed in stem than in other tissues; Bra020445 (corresponding to At5g57380 or VERNALIZATION INSENSITIVE 3, VIN3) was preferentially expressed in leaf than in other tissues. The group III includes 26 genes which were almost all very lowly (or not) expressed in the tested tissues, except for Bra028463 (corresponding to At5g40320, a cysteine/histidine-rich C1 domain family protein gene) preferentially expressed in callus but very lowly (or not) expressed in other tissues; Bra012982 (corresponding to At5g61090, a polynucleotidyl transferase gene) was preferentially expressed in silique, moderately expressed in flower, but very lowly (or not) expressed in other tissues; and Bra033990 (homologous to At2g21840, At2g21850 and At2g21830, cysteine/histidine-rich C1 domain family protein genes) was moderately but preferentially expressed in root.

**Fig. 6:**
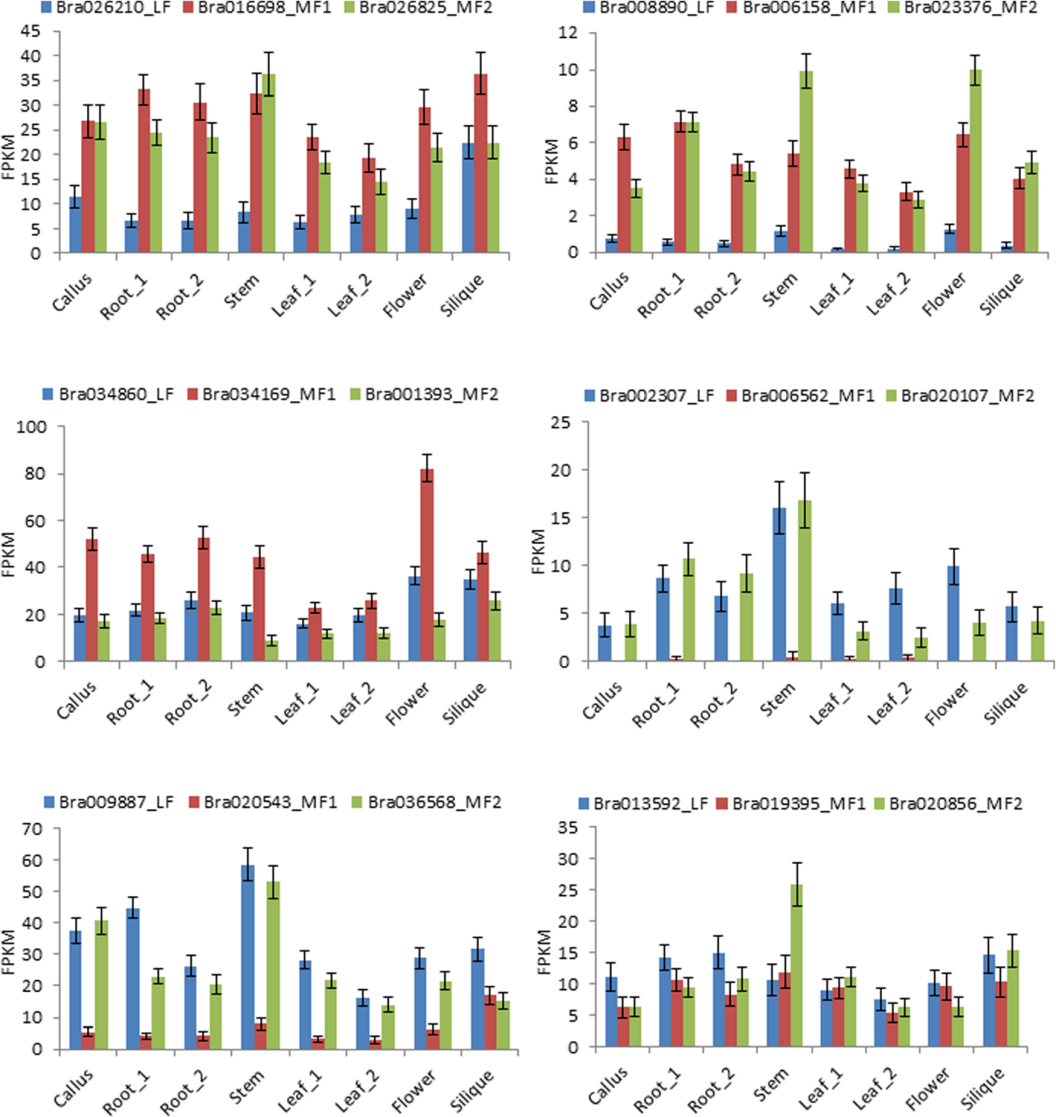
Comparison of the expression levels (by FPKM values) in different tissues between the triplicated *Brassica rapa* PHD finger gene members conserved across the three subgenomes LF, MF1 and MF2

To obtain information about the variation in expression pattern among triplicated PHD finger gene members caused by WGT [42, 71], we compared the expression levels (FPKM values) of six sets of three triplicated members that were well conserved across the three subgenomes (LF, MF1 and MF2) of *B. rapa* in different tissues (Fig. 6, Additional file 2: Table S5). The results showed that these triplicated members display different expression patterns between them. For four of six triplet sets, two members maintained relatively higher expression levels while the third one was significantly lowly expressed in the tested tissues. For one triplet set, one member showed a dominant high expression level over two other members in all tested tissues, while for another triplet set, one member was dominantly expressed over two other members in some tissues but not in others (Fig. 6).

Table 4 summarized the distribution of 140 PHD finger genes in different expression groups of Fig. 5 in relation to the phylogenetic classification of their encoded proteins in Fig. 2. We observed that 42.9% of genes in phylogenetic group A, 88.9% in group B, 70.4% in group C, 55.6% in group D, 56.7% in group E and 50.0% in group F were clustered into the expression group I (constitutively expressed in almost all the tested tissues). About 20% of genes in phylogenetic group A, 10% in group B, and 30% in group C, D, E and F were clustered into the expression group II (preferentially expressed in some tissues). About 40% of genes in phylogenetic group A, 0% in group B and C, 10% in group D and E, and 20% in group F were clustered into the expression group III (very lowly or not expressed in almost all the tested tissues).

**Table 4:** Distribution of 140 PHD finger genes in different expression groups of Fig. 5 in relation to the phylogenetic classification of their encoded proteins in Fig. 2. Values in parentheses indicate the percentages of genes per total genes of each phylogenetic group in each expression group

Expression group	No. of genes	Group A	Group B	Group C	Group D	Group E	Group F
I	78	21 (42.9)	8 (88.9)	19 (70.4)	5 (55.6)	17 (56.7)	8 (50.0)
II	36	9 (18.4)	1 (11.1)	8 (29.6)	3 (33.3)	10 (33.3)	5 (31.3)
III	26	19 (38.7)	0 (0.0)	0 (0.0)	1 (11.1)	3 (10.0)	3 (18.7)
Total No.	140	49	9	27	9	30	16

### Expression analysis of *B. rapa* PHD finger genes under salt and drought stresses

**Fig. 7:**
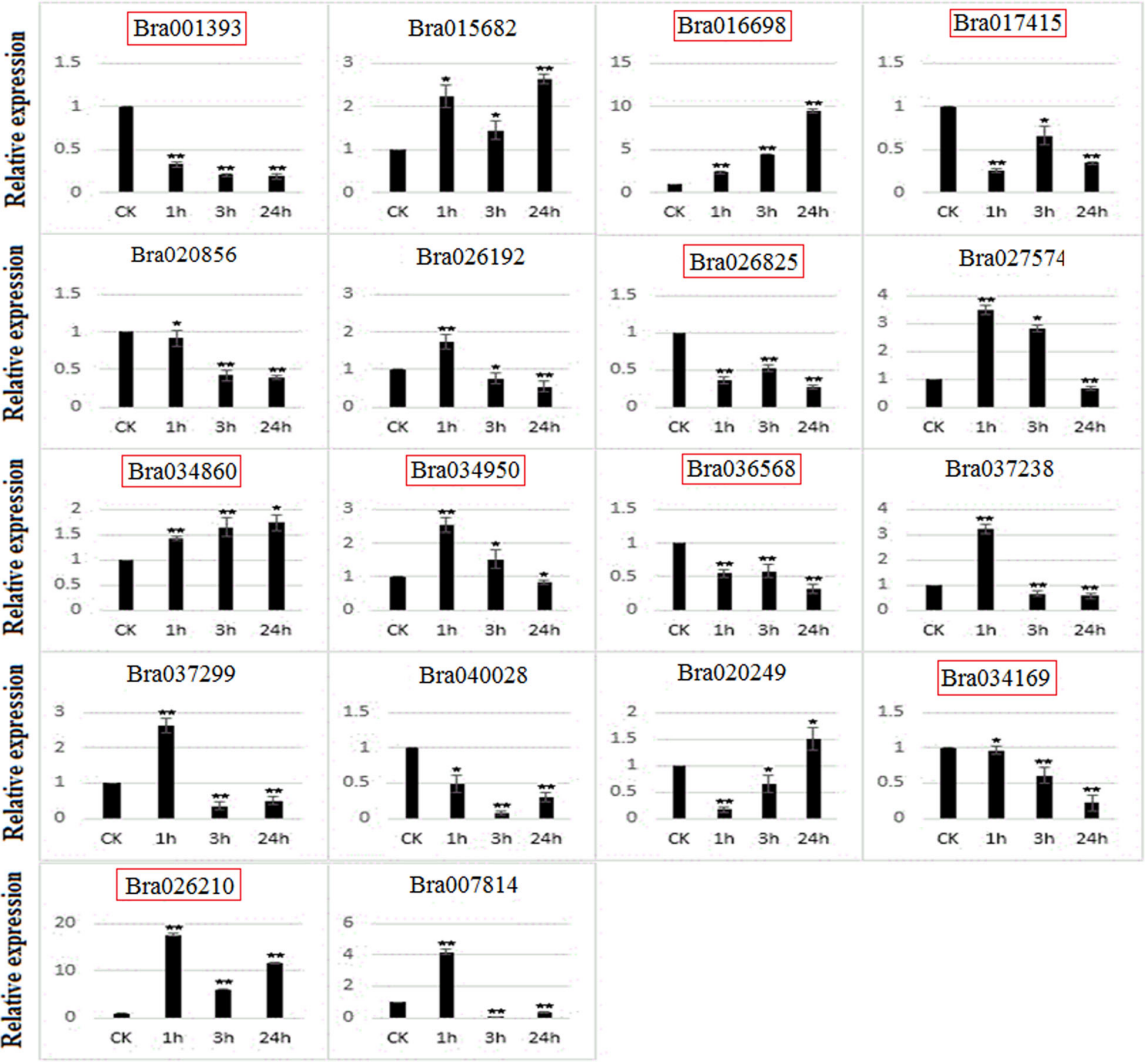
qRT-PCR expression patterns of 18 *Brassica rap* PHD finger genes under salt treatment. The time points represent by x-axis and the scale of relative expression showed by y-axis. Statistical significance of deference’s between control and treated groups was analyzed using Student’s t-test (*indicates *P* < 0.05, ** indicates *P* < 0.01). The “stress-related” genes (see the text) are framed by red box

**Fig. 8:**
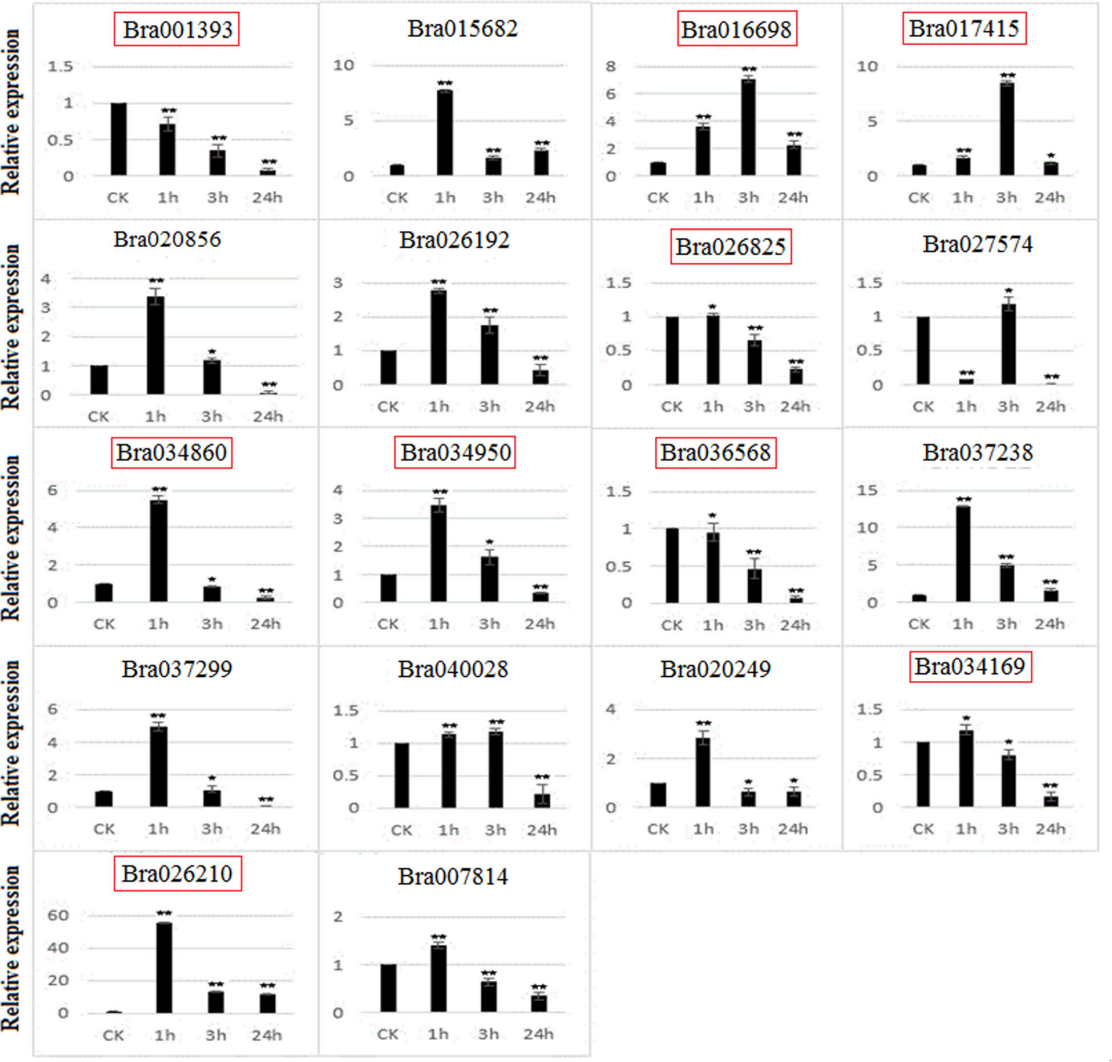
qRT-PCR expression patterns of 18 *Brassica rap* PHD finger genes under drought treatment. The time points represent by x-axis and the scale of relative expression showed by y-axis. Statistical significance of deference’s between control and treated groups was analyzed using Student’s t-test (*indicates *P* < 0.05, ** indicates *P* < 0.01). The “stress-related” genes (see the text) are framed by red box

In order to relate our results with the existing data from other species, we generated a phylogenetic tree by using the protein sequences of 145 *B. rapa* PHD finger genes together with those of a few previously reported as stress- or development-related in maize [64], poplar [65], soybean [61, 62], *alfalfa* [60], *Arabidopsis* [75–80] and rice [81–83] (Additional file 1: Figure S5). We found that 18 *B. rapa* PHD finger genes were clustered together with those previously characterized as stress-related, while 63 others were clustered together with those previously reported as development-related. Genes closely clustered together in a phylogenetic tree may share common ancestors, and their functions may be conserved across species. Based on this phylogenetic tree (Additional file 1: Figure S5), we selected nine genes (Bra001393, Bra016698, Bra017415, Bra026210, Bra026825, Bra034169, Bra034860, Bra034950 and Bra036568) representing the “stress-related”, and nine other genes (Bra007814, Bra015682, Bra020249, Bra020856, Bra026192, Bra027574, Bra037238, Bra037299, Bra040028) representing the “development-related” or non-characterized genes for qRT-PCR analysis to examine their expression response to salt (200 mM NaCl) (Fig. 7) and drought (20% (w/v) PEG_6000_) (Fig. 8) stresses in the leaves of three-week-old seedlings. Our results showed that all the selected 18 *B. rapa* PHD finger genes were responsive to the two abiotic stress treatments.

For salt stress analysis, all the 18 tested genes were responsive to the treatment with 11 PHD finger genes up-regulated and seven genes down-regulated compared to control (CK) after 1 h, 3 h or 24 h of treatment, respectively (Fig. 7). The most spectacular case is the gene Bra026210 (corresponding to At1g14510 or ALFIN-LIKE 7, AL7, involved in covalent chromatin modification or regulation of transcription) which was induced by more than 18 fold under salt treatment at 1 h. Interestingly, Bra016698, a paralogous copy of Bra026210 produced by WGT (Additional file 2: Table S5), was progressively induced along with the time under salt treatment and reached as high as nine fold of the control at 24 h, while another paralogous copy Bra026825 was down-regulated by more than two fold. Another gene, named Bra007814 (corresponding to At2g25170 or CYTOKININ-HYPERSENSITIVE 2, CKH2, involved in covalent chromatin modification and negative regulation of transcription) was up-regulated by four fold at 1 h of treatment but significantly down-regulated at 3 and 24 h.

For drought stress analysis, all the 18 tested genes were responsive to treatment with 16 genes up-regulated and two down regulated compared to control (CK) after 1 h, 3 h or 24 h of treatment, respectively (Fig. 8). Globally, the variation extents induced by drought stress were more spectacular than salt stress. Interestingly, Bra026210 was also highly induced by drought stress as it was the case for salt stress (Fig. 7), and reached an expression level of as high as 55 times compared to the control at 1 h, followed by an expression level of about 15 times of the control at 3 or 24 h. Bra037238 (corresponding to At2g18090, involved in regulation of transcription), Bra015682 (corresponding to At1g77250, involved in regulation of transcription) and Bra034860 (corresponding to At3g11200 or ALFIN-LIKE 2, AL2, involved in covalent chromatin modification or regulation of transcription) were induced by about 13, seven and five fold, respectively, compared to control at 1 h of treatment. Bra017415 (corresponding to At2g02470 or ALFIN-LIKE 6, AL6, involved in covalent chromatin modification or regulation of transcription) and Bra016698 (corresponding to At1g14510, or ALFIN-LIKE 7, AL7, involved in covalent chromatin modification or regulation of transcription) were induced by about eight and seven fold, respectively, compared to control at 3 h of treatment. It is worth to note that the three triplicated paralogous genes, Bra016698, Bra026210 and Bra026825, display different expression patterns along with the time of treatments of both salt and drought stress (Figs. 7) and (8).

## Discussion

PHD fingers can specifically recognize various histone marks or post-translational histone modifications (PTMs) such as trimethylated Lysine 4 in histone H3 (H3K4me3), trimethylated Lysine 9 in histone H3 (H3K9me3), trimethylated Lysine 36 in histone H3 (H3K36me3), acetylated Lysine 9 in histone H3 (H3K9ac), acetylated Lysine 14 in histone H3 (H3K14ac)*,* etc.*,* as well as unmodified histone tails such as H3K4, and other non-histone proteins [34–36, 84]*.* For example, all the PHD domains of the seven *Arabidopsis* Alfin1-like proteins (AL1 to AL7) can bind to the histone H3K4me3 peptide with varying methylation state preference and binding affinities [85]*;* rice CHD3 protein acts as a bifunctional chromatin regulator able to recognize and modulate H3K4 and H3K27 methylation over repressed or tissue-specific genes [86]; PHD finger of the SUMO ligase Siz/PIAS family in rice reveals specific binding for methylated histone H3 at lysine 4 and arginine 2 [87]. These features highlight the functional versatility of PHD fingers as epigenome readers that regulate gene expression (activation or repression) according to the status of the chromatin, and reinforce the hypothesis that evolutionary changes in amino acids surrounding the eight conserved metal ligand positions on a conserved structural fold would increase the functional diversity of these PHD finger proteins [35].

More and more plant PHD-finger protein genes have been identified as involved in various important biological processes. For examples, in the model plant *A. thaliana*, *MMD1* (AT1G66170), *SCC2* (AT5G15540), *MS1* (AT5G22260) and *ASHR3* (AT4G30860) are involved in the meiosis and post-meiotic processes, and their mutations can cause male sterility [36]; *OBE1* (AT3G07780), *OBE2* (AT5G48160) and *PKL* (AT2G25170) are involved in the embryonic meristem initiation and root development, and their mutations can result in an absence of root and defective development of the vasculature [36]; *AL6* (AT2G02470) and *AL7* (AT1G14510) are involved seed germination, and their double mutation can result in a germination delay under osmotic stress conditions [36]; *VIL1* (AT3G24440), *VRN2* (AT4G16845), *VIN3* (AT5G57380), *VRN5* (AT3G24440), *ATX1* (AT2G31650), *EBS* (AT4G22140) and *SHL* (AT4G39100) are involved in the control of flowering time [36]; *GSR1* (AT3G27490) is involved in auxin-mediated seed dormancy and germination [88]; *EDM2* (AT5G55390) is involved the resistance to downy mildew [89]; *AL5* (AT5G20510) is involved in abiotic stress tolerance [63]. In rice, *Ehd3* acts as a promoter in the unique genetic pathway responsible for photoperiodic flowering [81]; *PTC1* is involved in tapetal cell death and pollen development [83]; *OsVIL2* is involved in the control of flowering time, and its insertion mutations cause late flowering under both long and short days [90]; *OsTTA* is a constitutively expressed regulator of multiple metal transporter genes responsible for essential metals delivery to shoots for their normal growth [91]; *OsMS1* functions as a transcriptional activator to regulate programmed tapetum development and pollen exine formation [92]. In barley, *HvMS1* silencing and overexpression can result in male sterility [93]. In maize, the mutation of *ZmMs7* (ortholog of *PTC1*) can result in male sterility [94]. In soybean, all six Alfin1-type PHD finger genes were found to be responsive to various stress treatments, and overexpressing the *GmPHD2* showed salt tolerance when compared with the wild type plants [61]; *GmPHD5* encodes an important regulator for crosstalk between histone H3K4 di-methylation and H3K14 acetylation in response to salinity stress [62]. In alfalfa, *Alfin1* is involved in salt tolerance [59, 60]. In cassava, *MePHD1* is involved in starch synthesis [95]. Although the number of identified PHD-finger genes is increasing in different species, most of putative PHD finger genes remain to be characterized, and no any research on this category of genes has been conducted in *Brassica* species *to this day*.

*B. rapa* (AA genome) is not only an economically important vegetal, oilseed and fodder crop widely grown around the world, but also one of the diploid progenitor parents of amphidiploid oilseed crops *B. napus* (AACC) and *B. juncea* (AABB), and can be used as a model plant for functional and evolutionary studies of important gene families among *Brassica* species [49]. In this study, a total of 145 PHD finger proteins containing 233 PHD domains were identified from the current version of the *B. rapa* proteome database (Additional file 2: Table S1). This number is considerably higher than those previously identified in maize (67) [64], poplar (73) [65] and rice (59) [66, 96], pear (31) [97] and moso bamboo (60) [67], although it might not yet be exhaustive as other PHD-suspected domain-containing proteins were also detected (Additional file 2: Table S2). This is the consequence of the WGT event occurred ~ 15.9 MYA in Brassica ancestor followed by gene losing [42, 47, 71], while only one tandem duplication event was observed among these PHD finger genes (Fig. 4). Interestingly, these PHD finger genes were unevenly distributed on the 10 B. rapa chromosomes (Fig. 2), a phenomenon also observed in *A. thaliana* [8], maize [64] [65] and rice [66], implying a possible relationship between chromosomal location and their cellular functions.

Our gene ontology analysis showed that 67.7% of the identified *B. rapa* PHD finger proteins were predicted to be located in nucleus, and 91.3% members were putatively involved in protein binding activity (Fig. 1). These features support the previous findings about the main functions of these PHD finger genes as epigenomic effectors regulating gene expression in cells [30–36]. Based on the presence or not of additional domains (Additional file 2: Table S4), gene structure and phylogenic analysis (Figs. 2 and 3; Additional file 1: Figure S2), these *B. rapa* PHD finger genes can be classified into several groups and subgroups, illustrating the evolution and functional diversification of these genes in *B. rapa*. Our phylogenetic (Fig. 2) and syntenic (Additional file 2: Table S5) analyses showed that, for the majority of *B. rapa* PHD finger genes, their corresponding orthologs were also found in the model plant *A. thaliana*, meant that the functional study of *B. rapa* PHD finger genes can largely benefit from the rich information available in *A. thaliana* (Additional file 2: Table S3). However, as shown in Fig. 2, the duplicated gene members in *B. rapa* generally evolved at different rates in comparison with their orthologs in *A. thaliana*, furthermore, some *B. rapa* finger protein genes, such as Bra038151 and Bra029800 in phylogenetic group A, Bra026192 in group B, and Bra034957 in group C, * etc*., cannot find their corresponding orthologs in *A. thaliana*, suggesting that these genes may provide some new or specific functions for the growth and development of *Brassica* crops or their responses to various stresses.

Our analysis on RNA-Seq data (Fig. 5) showed that 55.7% of the *B. rapa* PHD finger genes were constitutively expressed in all the tested tissues with relatively higher expression levels, suggesting their important housekeeping roles in plant growth and development. A few PHD finger genes were also identified as preferentially expressed in specific tissues, constituting then an interesting panel of candidates for future targeted studies on the function of PHD finger genes and genetic improvement of *Brassica* species. Comparison of expression levels between triplicated members (Fig. 6) showed that in the majority of cases the three triplicated members display varied expression levels and patterns across different tissues, indicating that their biological roles may be also varied in plant growth and development, a phenomenon of neo-functionalization or sub-functionalization of duplicated genes [98]. Existence of 1–2 triplicated members that display a very low (or not) expression level contrasting to the higher expression levels of other triplicated members of the same triplet set, such as the case of Bra006562 in Fig. 6, indicates that they may be degenerated during the evolution, a phenomenon already observed previously for RING finger protein genes [99].

In this study, we also analyzed the expression patterns of 18 *B. rapa* PHD finger genes in response to drought and salt stresses, of which nine have been characterized previously as “stress-related” in other plant species [60–62, 64, 65], while nine other genes representing the “development-related” or non-characterized genes (Additional file 1: Figure S5). Our results showed that all these genes were responsive to the two abiotic stress treatments with different amplitudes and varied expression patterns: some members were highly up-regulated while others were down-regulated along with the time of treatments (Fig. 7, Fig. 8). This means that some PHD finger genes may play important roles in plant adaption to adverse environmental stresses, an idea that was also supported by other studies [61, 64–66]. These identified PHD finger genes can then serve as a source of candidate genes for genetic engineering and improvement of *Brassica* crops against abiotic stresses. Further studies extended on other PHD finger genes with more types of abiotic stress treatments would allow us to obtain a global view on the involvement of these PHD finger genes in response to abiotic stresses, and identify the most prominent ones for use as targets in genetic improvement of stress resistance in plants.

## Conclusions

We identified a total of 145 putative PHD finger proteins containing 233 PHD domains from the current release of *B. rapa* genome database. These PHD finger genes were further characterized and classified into different groups or categories by analyses of gene ontology, additional domain, gene structure, synteny and phylogeny. We also analyzed the RNA-Seq data of these PHD finger genes, and found that 55.7% of them were constitutively expressed in all the tested tissues with relatively higher expression levels. Expression analysis of a subset of 18 PHD finger genes under salt and drought treatments showed that all of them were responsive to the two abiotic stresses, indicating that PHD finger genes can be a source of candidate genes for genetic improvement of *Brassica* crops against abiotic stresses. Our results lay the foundation for further functional determination of each PHD finger gene across the *Brassica* species, and may help to select the most promising gene targets for further genetic engineering and improvement of *Brassica* crops.

## Methods

### Identification and characterization of PHD finger proteins in *B. rapa*

To identify all *B. rapa* PHD finger proteins, we followed two different strategies as have been described in a previously study [50]. First, all previously identified *Arabidopsis* PHD finger proteins [96, 100] were used as query sequences for BLASTp searches against the *B. rapa* proteome database at BRAD (http://brassicadb.org/brad/). Second, all *Arabidopsis* PHD finger domains as well as those of maize [64] and poplar [65] were used as query sequences for BLASTp searches against the same *B. rapa* proteome database at BRAD. The irredundant candidate sequences were then analyzed online by SMART (http://smart.embl-heidelberg.de) (option Pfam) and occasionally by InterPro (http://www.ebi.ac.uk/interpro/) to confirm the presence or not of PHD domains. This was followed by visual inspections based on the conservation of eight metal ligands (Cys4-His-Cys3) and the residue number between two neighboring metal ligands, especially between the 4th and 5th metal ligands where the number of residues should be four or five for a PHD domain contrasting to two or three for RING and two for LIM [13]. Those proteins that were predicted as PHD domain-containing by SMART but lacked two or more metal ligands, or those containing a sequence motif visually resembled to a PHD domain but not validated by SMART were classified as PHD-suspected domain-containing. The protein size, molecular weight (MW), and theoretical isoelectric point (pI) of each PHD finger protein were computed by using Pepstats (http://www.ebi.ac.uk/Tools/seqstats/emboss_pepstats/).

For each identified *B. rapa* PHD finger protein, their associated Gene Ontology (GO) terms were retrieved from the Phytozome database (http://phytozome.jgi.doe.gov/pz/portal.html), and their subcellular localization was predicted by CELLO v2.5 software (http://cello.life.nctu.edu.tw).

### Multiple sequence alignment, gene structure and phylogenetic analysis

The PHD finger domains were aligned by Clustal W and manually edited by BioEdit software. The sequence logo of over-represented motif among the identified PHD domains was generated by using the Web Logo software (http: //weblogo.berkeley.edu/logo.cgi). Phylogenetic trees based on *B. rapa* PHD finger domain sequences and the PHD finger protein sequences of *B. rapa* and *A. thaliana* were generated by using MEGA6.06 software with the Neighbor–Joining (NJ) algorithm and a bootstrap analysis of 1000 replicates. The exon/intron structure of each *B. rapa* PHD finger gene was generated by using the Gene Structure Display Server 2.0 (http://gsds.cbi.pku.edu.cn/).

### Additional domain analysis

To identify additional known domains, each predicted *B. rapa* PHD finger protein was analyzed by Smart (http:// smart.embl-heidelberg.de) with option Pfam. According to the presence and organization of different known domains, these *B. rapa* PHD finger proteins were divided into different groups. These additional domains were then used as query sequences for BLASTp searches against the NCBI database to determine if they were also present in other plant species.

### Chromosome location of PHD-finger protein genes in *B. rapa*

For chromosome mapping of the identified *B. rapa* PHD finger genes, we followed the same procedure that was described in our previous study [50]. For each putative PHD-finger protein gene, their physical chromosome location data were retrieved from the BRAD database. The Map Chart 2.3v software was used for mapping analysis.

### Syntenic relationships between *B. rapa* and *A. thaliana* PHD finger genes

For establishing the syntenic relationships among the identified *B. rapa* PHD finger genes, we followed the same procedure that was described previously [50]. The Search Syntenic Gene function of the BRAD database was used to find out the syntenic paralogs in *B. rapa* and orthologs in *A. thaliana*. The information such as gene name (s), localization on ancestral chromosome blocks of the tPCK (Translocation Proto-Calepineae Karyotype), *Arabidopsis* chromosomes and *B. rapa* LF, MF1 and MF2 subgenomes [43–45, 72], as well as the possible tandem repeats in the two species, were recorded and summarized in Additional file 2: Table S5.

### Expression pattern of PHD finger genes in *B. rapa*

The RNA-Seq data of six tissues (callus, root, stem, leaf, flower and silique) of the *B. rapa* accession Chiifu-401–42 was downloaded from NCBI (http://www.ncbi.nlm.nih.gov/geo/) (GEO accession GSE43245) [74]. For each identified *B. rapa* PHD finger gene, their expression values (Fragments Per Kilobase of exon model per Million mapped, FPKM) of were extracted from the data set. The clustering analysis was then conducted by using Cluster software v3.0 (http://bonsai.hgc.jp/~mdehoon/software/cluster/) with the options of log2-transformed, Euclidean distances and the average linkage clustering method. The Java Tree view software (http://jtreeview.sourceforge. net/) was used to generate a clustering gene expression heatmap.

### Plant materials and stress treatments

For preparation of plant materials and stress treatments, we followed the same procedures that were described in our previous paper [50].* B. rapa* accession Chiifu-401–42 seeds were first germinated in a Petri dish at 25 °*C,* then transferred into plastic pots in a greenhouse at *22* °*C* with *16/8 h* for light/dark. Stress treatments were conducted on 21-days-old seedlings., The plants were irrigated with 200 mM NaCl and 20% (w/v) polyethylene glycol (PEG 6000) for salt and drought stress treatments, respectively. For each treatment, three biological replicates were prepared. The leaves from control and stressed plants were harvested in liquid nitrogen after 0, 1, 3, and 24 h of treatments, and placed at − 80 °C before RNA extraction.

### RNA isolation and quantitative real-time PCR (qRT-PCR) analysis

For RNA isolation and quantitative real-time PCR (qRT-PCR) analysis, we followed the same procedures that were described in a previous study [50]. Total RNA was isolated from approximately 100 mg of the frozen leaves of each sample using an OMEGA Plant RNA extraction Kit. RNA concentrations were estimated by using a NanoDrop 2000 Spectrophotometer (Thermo Fisher Scientific, Inc., USA). First-strand cDNA was synthesized by using a TaKaRa cDNA Synthesis Kit (Dalian, China). Gene-specific primers were designed using the online Primer3Plus software (http://www.primer3plus.com/). The *B. rapa Actin*-2 gene (XM_018658258) was used as internal reference gene. The primers used in this study were presented in Additional file 2: Table S6. The qRT-PCR analysis was conducted on an ABI 7500 Fast Real-time PCR amplification system (Applied Biosystems, USA) in a volume of 20 μL: 2 μL cDNA template, 0.8 μL forward primers (10 μM), 0.8 μL reverse primers (10 μM), 10 μL SYBR Green PCR Master (ROX) (Roche, China), and 6.4 μL sterile water. The amplification parameters were: 95 °C for 1 min, followed by 40 cycles of 95 °C for 15 s, and 60 °C for 70 s. The 2^−ΔΔCt^ method [101] was usd for data analysis. The Student’s t-test was used to determine the significance of differences among relative expression levels of each tested gene at different time points of treatment (with *P* < 0.05 considered as statistically significant).

## Supplementary information

**Additional file 1: Fig. S1.** Multiple sequence alignment of 233 PHD domains from 145 PHD finger proteins of *Brassica rapa.* **Fig. S2.** Sequence logo of the overrepresented motif found in 233 PHD domains of *Brassica rapa*. **Fig. S3.** Phylogenetic tree based on multiple sequence alignment of 233 PHD domains from 145 putative PHD finger proteins of *Brassica rapa*. **Fig. S4.** Phylogenetic tree based on multiple sequence alignment of PHD finger proteins from *Arabidopsis thaliana*, *Brassica rapa*, *Oryza sativa*, *Populus trichocarpa *and *Zea mays.* **Fig. S5.** Phylogenetic tree analyses of all 145 *Brassica rapa* PHD finger proteins and a few PHD finger proteins from other species previously characterized as stress or plant development related.

**Additional file 2: Table S1.** List of 145 *B. rapa* PHD finger protein genes, and their related informations**. Table S2**. List of 16 suspected *B. rapa* PHD finger proteins. **Table S3.** Summary of gene ontology terms of 98 *A. thaliana* PHD finger protein genes (retrieved from TAIR database, https://www.arabidopsis.org/index.jsp) in relation to the phylogenetic classification of their encoded proteins along with the 145 *B. rapa* PHD finger proteins in Fig. 2. **Table S4.** Classification of145 *B. rapa* PHD domain-containing proteins based on the presence or not and organization of additional domain (s). **Table S5.** Synteny relationships between *Arabidopsis* and *B.rapa* PHD finger protein genes. **Table S6.** The information of primers used in the quantitative real-time PCR (qRT-PCR) analysis.

## Data Availability

Not applicable.

## Abbreviations

FPKM: Fragments Per Kilobase of exon model per Million mapped
GEO: Gene Expression Omnibus
LF: Least fractionized subgenome
LIM: Lin-ll, Isl-1 and Mec-3
MF1: Moderately fractionized subgenome
MF2: Most fractionized subgenome
MW: Molecular weight
MYA: Million years ago
PEG: Polyethylene glycol
PHD: Plant homeodomain
pI: Theoretical isoelectric point
qRT-PCR: quantitative real-time PCR
RING: Really interesting new gene
RNA-Seq: RNA sequencing;
TFIIIA: Transcription factor IIIA
tPCK: translocation Proto-Calepineae Karyotype
WGT: Whole genome triplication
