# Supplementary material for: Genome wide survey, evolution and expression analysis of PHD finger genes reveal their diverse roles during the development and abiotic stress responses in Brassica rapa L
Source: BMC Genomics. 2019 Oct 24;20:773. doi: 10.1186/s12864-019-6080-8 (PMC6814106; doi:10.1186/s12864-019-6080-8)

Supplementary figures

Fig. S1 Multiple sequence alignment of 233 PHD domains from 145 PHD finger proteins of *Brassica rapa*.

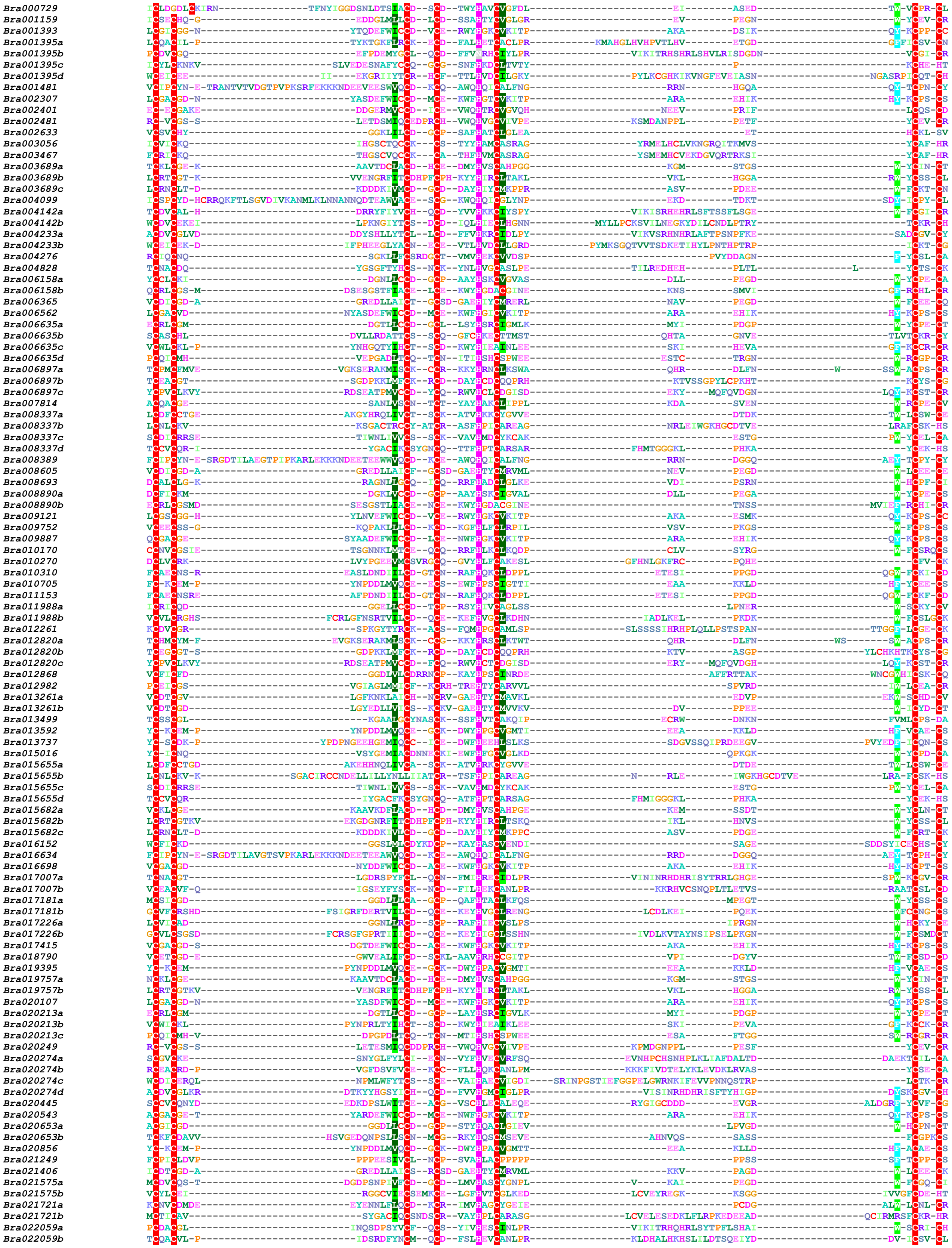

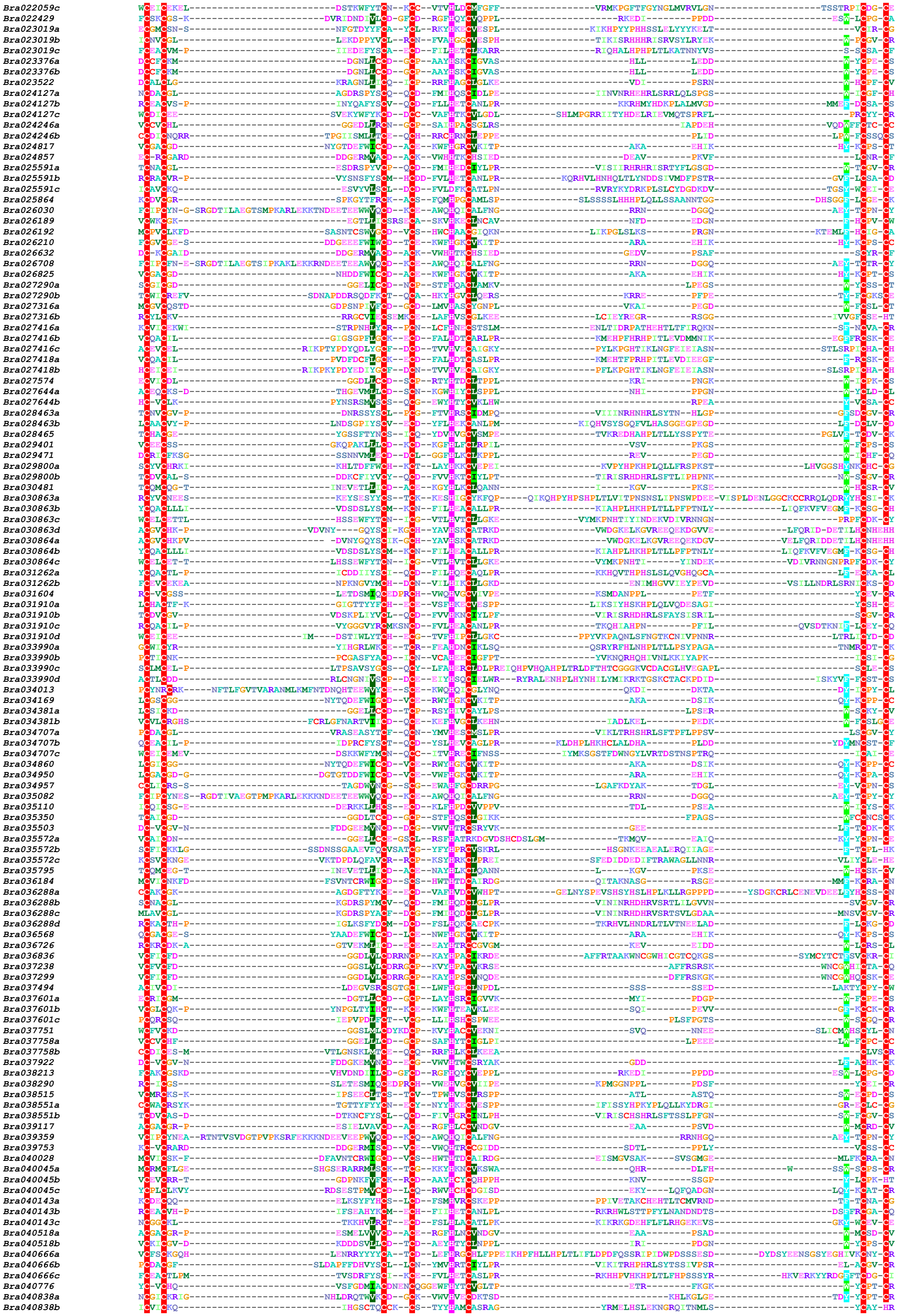
Bra022059c
Bra022429
Bra023019a
Bra023019b
Bra023019c
Bra023376a
Bra023376b
Bra023522
Bra024127a
Bra024127b
Bra024127c
Bra024246a
Bra024246b
Bra024817
Bra024857
Bra025591a
Bra025591b
Bra025591c
Bra025864
Bra026030
Bra026189
Bra026192
Bra026210
Bra026632
Bra026708
Bra026825
Bra027290a
Bra027290b
Bra027316a
Bra027316b
Bra027416a
Bra027416b
Bra027416c
Bra027418a
Bra027418b
Bra027574
Bra027644a
Bra027644b
Bra028463a
Bra028463b
Bra028465
Bra029401
Bra029471
Bra029800a
Bra029800b
Bra030481
Bra030863a
Bra030863b
Bra030863c
Bra030863d
Bra030864a
Bra030864b
Bra030864c
Bra031262a
Bra031262b
Bra031604
Bra031910a
Bra031910b
Bra031910c
Bra031910d
Bra033990a
Bra033990b
Bra033990c
Bra033990d
Bra034013
Bra034169
Bra034381a
Bra034381b
Bra034707a
Bra034707b
Bra034707c
Bra034860
Bra034950
Bra034957
Bra035082
Bra035110
Bra035350
Bra035503
Bra035572a
Bra035572b
Bra035572c
Bra035795
Bra036184
Bra036288a
Bra036288b
Bra036288c
Bra036288d
Bra036568
Bra036726
Bra036836
Bra037238
Bra037299
Bra037494
Bra037601a
Bra037601b
Bra037601c
Bra037751
Bra037758a
Bra037758b
Bra037922
Bra038213
Bra038290
Bra038515
Bra038551a
Bra038551b
Bra039117
Bra039359
Bra039753
Bra040028
Bra040045a
Bra040045b
Bra040045c
Bra040143a
Bra040143b
Bra040143c
Bra040518a
Bra040518b
Bra040666a
Bra040666b
Bra040666c
Bra040776
Bra040838a
Bra040838b

Fig. 2 Sequence logo of the overrepresented motif found in 233 PHD domains of *Brassica rapa*. The asterisked letters of Cys and/or His indicate conserved metal ligands. The zinc-coordinating amino acid pairs are shown. The figures were created by on-line Web Logo tool (http://weblogo.berkeley.edu/logo.cgi). The height of the letters is proportional to their frequency of the corresponding amino acid at that position.

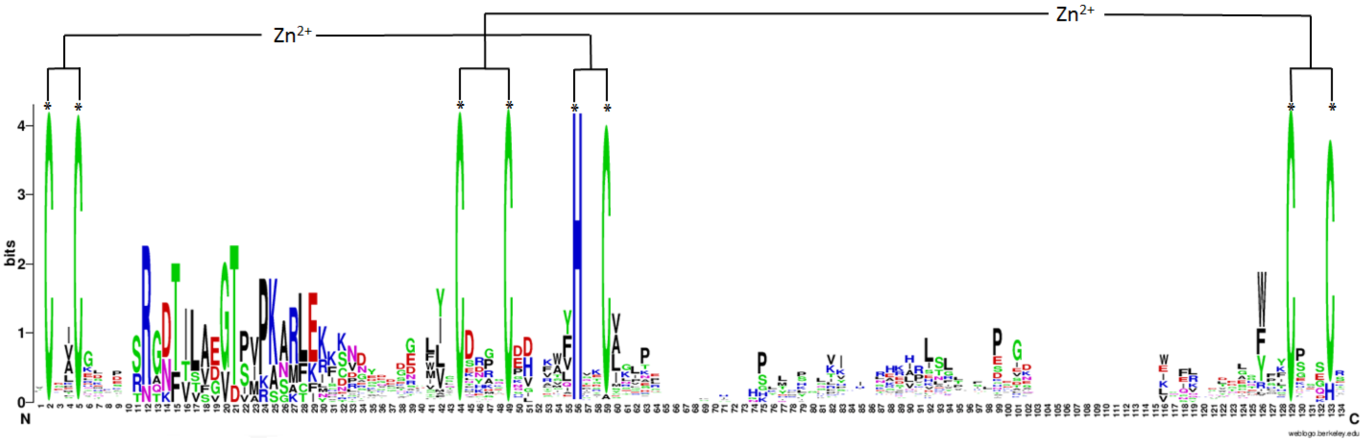

Fig. S3 Phylogenetic tree based on multiple sequence alignment of 233 PHD domains from 145 putative PHD finger proteins of *Brassica rapa*.

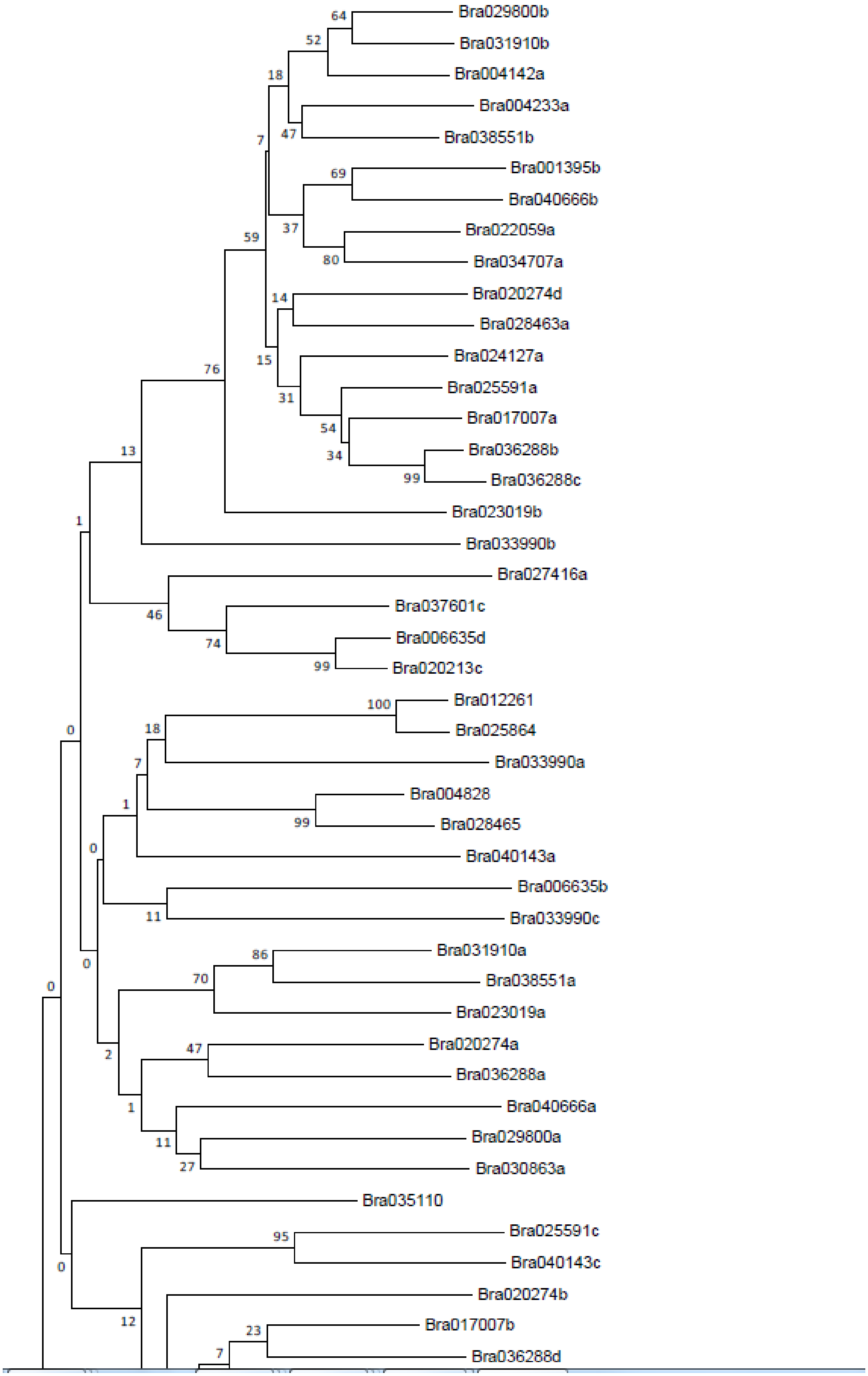

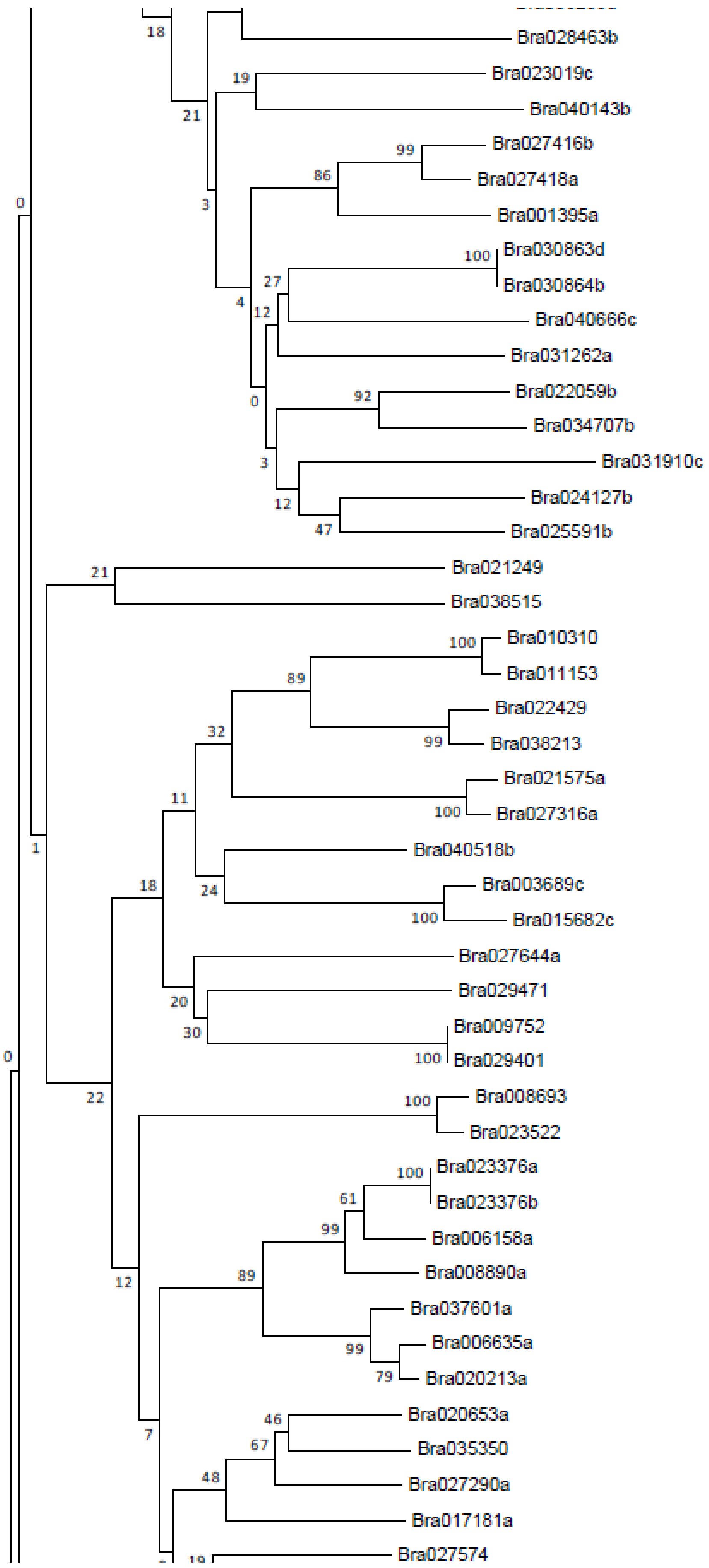
Bra028463b
Bra023019c
Bra040143b
Bra027416b
Bra027418a
Bra001395a
Bra030863d
Bra030864b
Bra040666c
Bra031262a
Bra022059b
Bra034707b
Bra031910c
Bra024127b
Bra025591b
Bra021249
Bra038515
Bra010310
Bra011153
Bra022429
Bra038213
Bra021575a
Bra027316a
Bra040518b
Bra003689c
Bra015682c
Bra027644a
Bra029471
Bra009752
Bra029401
Bra008693
Bra023522
Bra023376a
Bra023376b
Bra006158a
Bra008890a
Bra037601a
Bra006635a
Bra020213a
Bra020653a
Bra035350
Bra027290a
Bra017181a
Bra027574

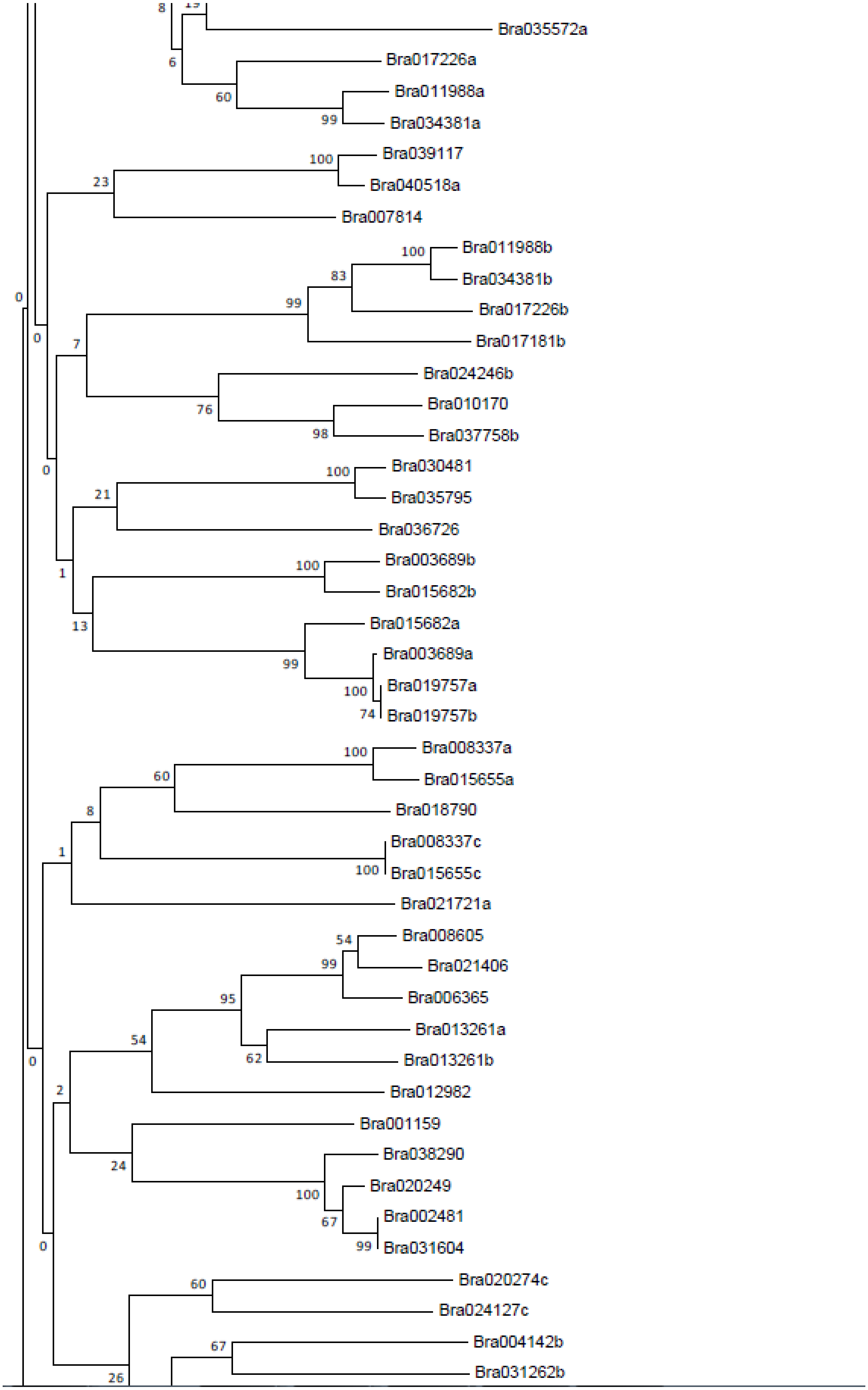
Bra035572a
Bra017226a
Bra011988a
Bra034381a
Bra039117
Bra040518a
Bra007814
Bra011988b
Bra034381b
Bra017226b
Bra017181b
Bra024246b
Bra010170
Bra037758b
Bra030481
Bra035795
Bra036726
Bra003689b
Bra015682b
Bra015682a
Bra003689a
Bra019757a
Bra019757b
Bra008337a
Bra015655a
Bra018790
Bra008337c
Bra015655c
Bra021721a
Bra008605
Bra021406
Bra006365
Bra013261a
Bra013261b
Bra012982
Bra001159
Bra038290
Bra020249
Bra002481
Bra031604
Bra020274c
Bra024127c
Bra004142b
Bra031262b

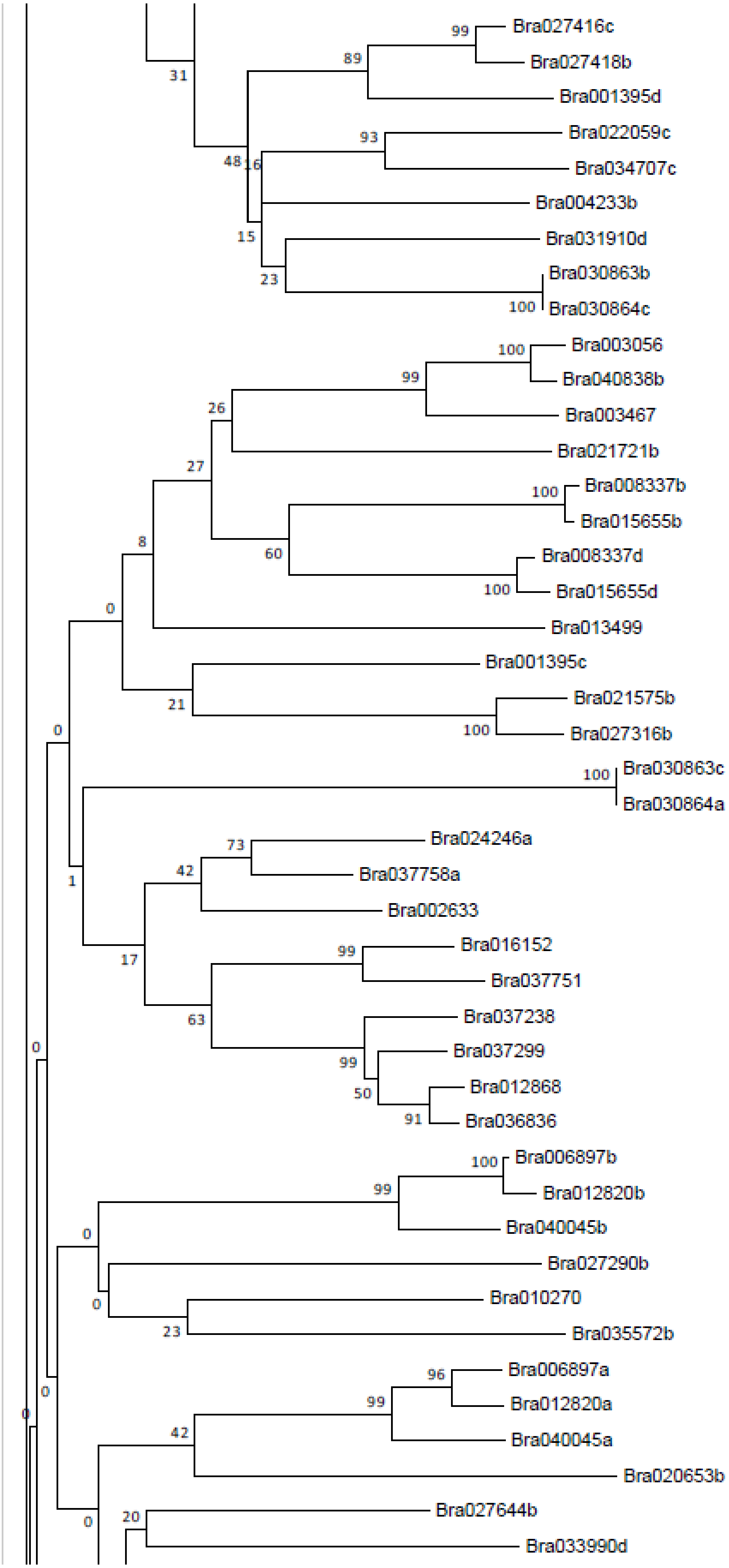
Bra027416c
Bra027418b
Bra001395d
Bra022059c
Bra034707c
Bra004233b
Bra031910d
Bra030863b
Bra030864c
Bra003056
Bra040838b
Bra003467
Bra021721b
Bra008337b
Bra015655b
Bra008337d
Bra015655d
Bra013499
Bra001395c
Bra021575b
Bra027316b
Bra030863c
Bra030864a
Bra024246a
Bra037758a
Bra002633
Bra016152
Bra037751
Bra037238
Bra037299
Bra012868
Bra036836
Bra006897b
Bra012820b
Bra040045b
Bra027290b
Bra010270
Bra035572b
Bra006897a
Bra012820a
Bra040045a
Bra020653b
Bra027644b
Bra033990d

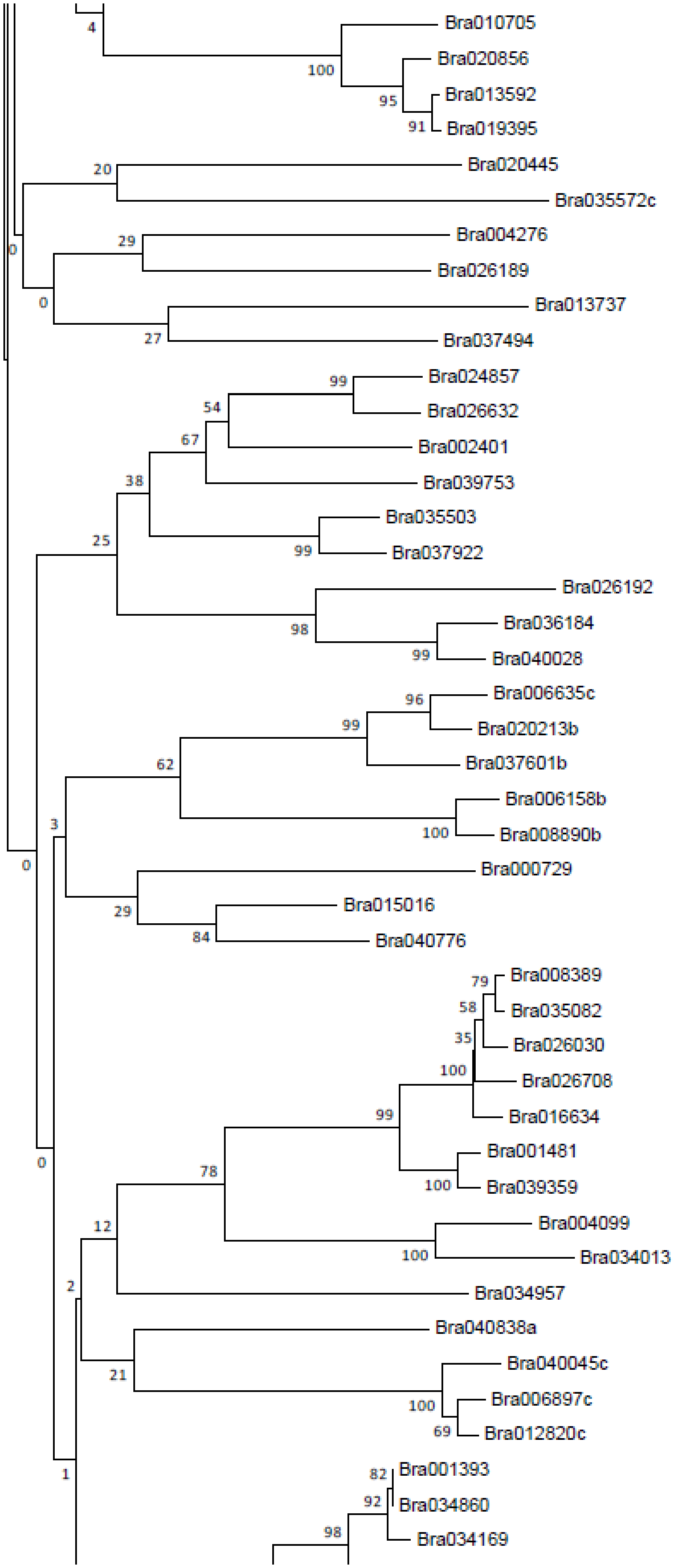

Bra010705
4
100
Bra020856
95
Bra013592
91
Bra019395
20
Bra020445
Bra035572c
29
Bra004276
0
Bra026189
0
Bra013737
27
Bra037494
99
Bra024857
54
Bra026632
67
Bra002401
38
Bra039753
Bra035503
25
99
Bra037922
Bra026192
98
Bra036184
99
Bra040028
96
Bra006635c
99
Bra020213b
62
Bra037601b
3
Bra006158b
100
Bra008890b
0
Bra000729
29
Bra015016
84
Bra040776
79
Bra008389
58
Bra035082
35
Bra026030
100
Bra026708
99
Bra016634
Bra001481
0
78
100
Bra039359
Bra004099
12
100
Bra034013
2
Bra034957
Bra040838a
21
Bra040045c
100
Bra006897c
69
Bra012820c
1
82
Bra001393
92
Bra034860
98
Bra034169

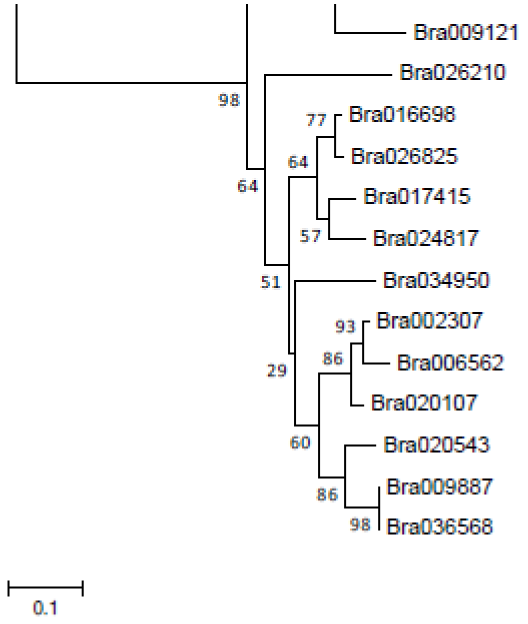
Bra009121
Bra026210
98
77
Bra016698
Bra026825
64
64
Bra017415
57
Bra024817
51
Bra034950
93
Bra002307
86
Bra006562
29
Bra020107
60
Bra020543
86
Bra009887
98
Bra036568
0.1

Fig. S4 Phylogenetic tree based on multiple sequence alignment of PHD finger proteins from *Arabidopsis thaliana*, *Brassica rapa*, *Oryza sativa*, *Populus trichocarpa* and *Zea mays*. The PHD finger proteins of *O. sativa*, *P. trichocarpa* and *Z. mays* were obtained according to the following the publications: (1) Sun M, Jia B, Yang J, Cui N, Zhu Y, Sun X. Genome-wide identification of the PHD-finger family genes and their responses to environmental stresses in *Oryza sativa* L. Int J Mol Sci. 2017;18(9):2005. (2) Wu S, Wu M, Dong Q, Jiang H, Cai R, Xiang Y. Genome-wide identification, classification and expression analysis of the PHD-finger protein family in *Populus trichocarpa*. Gene. 2016;575(1):75-89. (3) Wang Q, Liu J, Wang Y, Zhao Y, Jiang H, Cheng B. Systematic analysis of the maize PHD-finger gene family reveals a subfamily involved in abiotic stress response. Int J Mol Sci. 2015;16(10):23517-44.

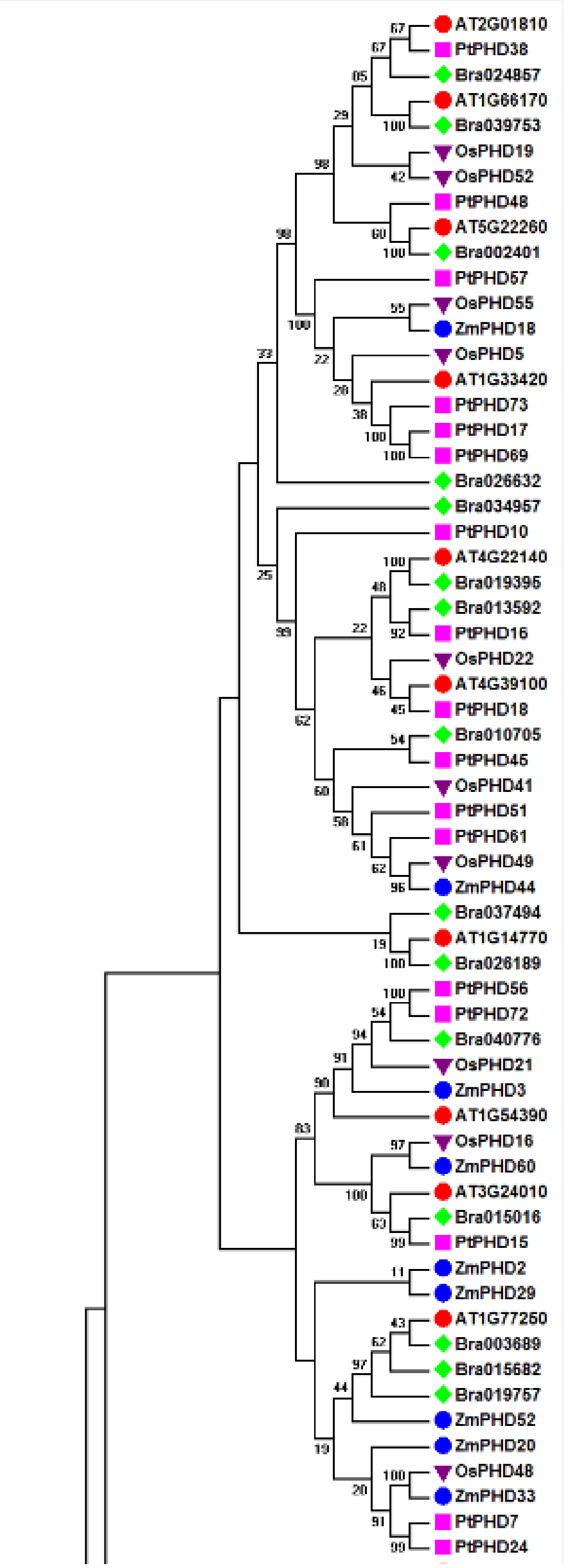

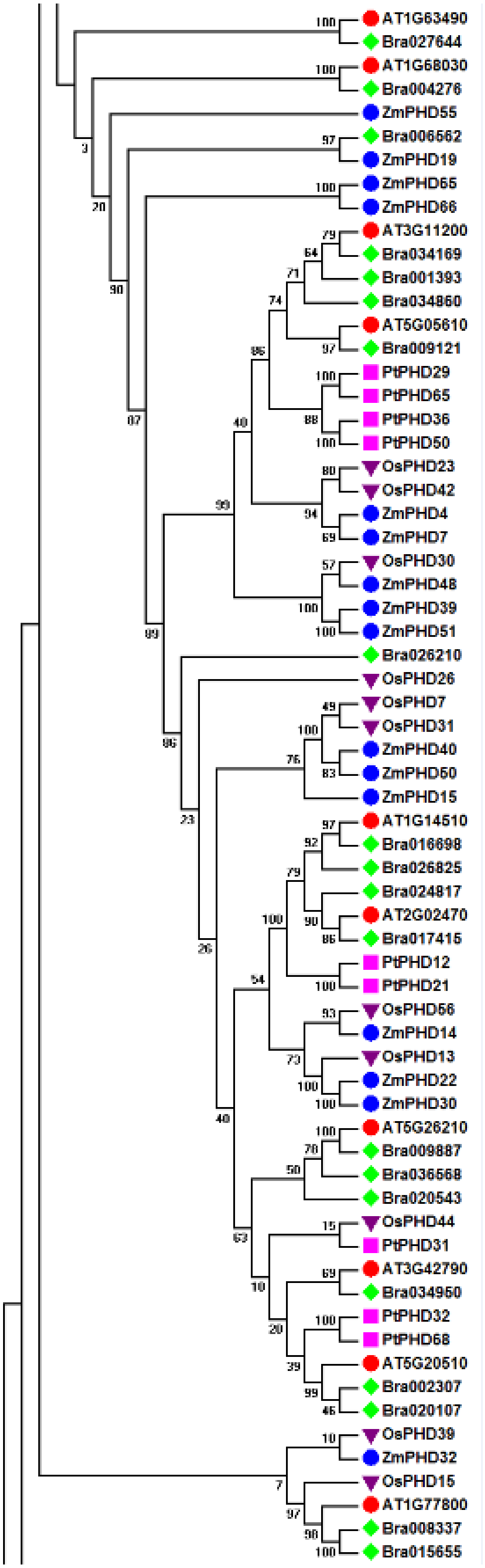
AT1G63490
Bra027644
AT1G68030
Bra004276
ZmPHD55
Bra006562
ZmPHD19
ZmPHD65
ZmPHD66
AT3G11200
Bra034169
Bra001393
Bra034860
AT5G05610
Bra009121
PtPHD29
PtPHD65
PtPHD36
PtPHD50
OsPHD23
OsPHD42
ZmPHD4
ZmPHD7
OsPHD30
ZmPHD48
ZmPHD39
ZmPHD51
Bra026210
OsPHD26
OsPHD7
OsPHD31
ZmPHD40
ZmPHD60
ZmPHD15
AT1G14510
Bra016698
Bra026825
Bra024817
AT2G02470
Bra017415
PtPHD12
PtPHD21
OsPHD56
ZmPHD14
OsPHD13
ZmPHD22
ZmPHD30
AT5G26210
Bra009887
Bra036668
Bra020543
OsPHD44
PtPHD31
AT3G42790
Bra034960
PtPHD32
PtPHD68
AT5G20510
Bra002307
Bra020107
OsPHD39
ZmPHD32
OsPHD15
AT1G77800
Bra008337
Bra015655

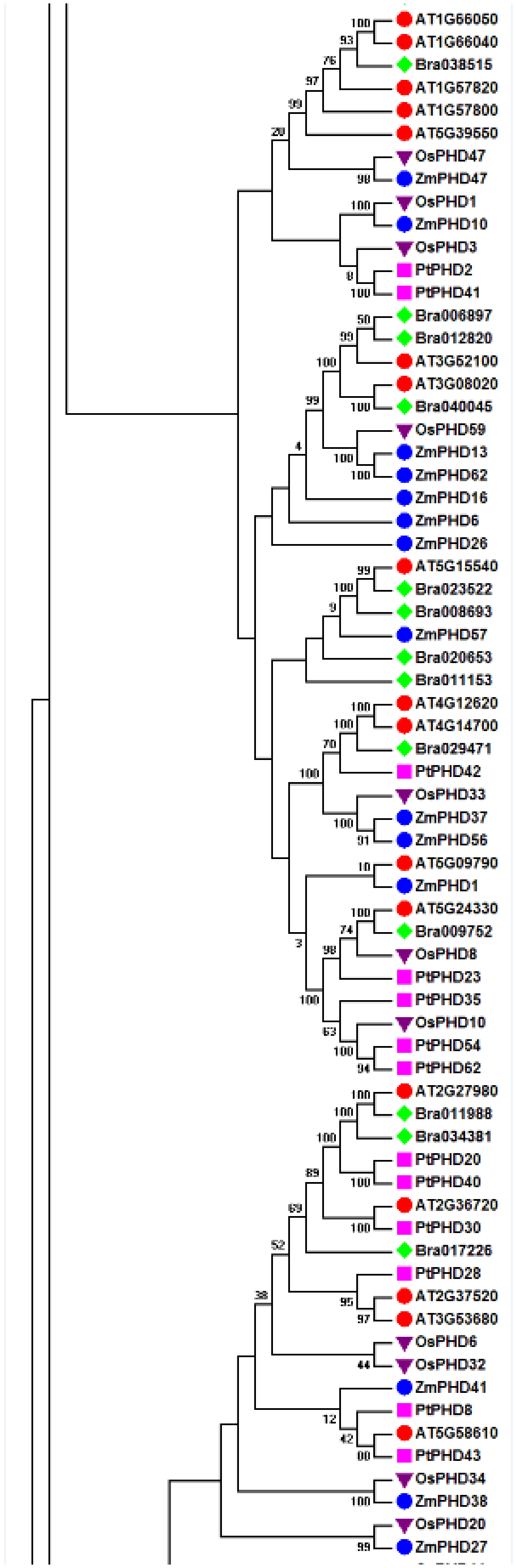
AT1G66050
AT1G66040
Bra038515
AT1G57820
AT1G57800
AT5G39550
OsPHD47
ZmPHD47
OsPHD1
ZmPHD10
OsPHD3
PtPHD2
PtPHD41
Bra006897
Bra012820
AT3G52100
AT3G08020
Bra040045
OsPHD59
ZmPHD13
ZmPHD62
ZmPHD16
ZmPHD6
ZmPHD26
AT5G15540
Bra023522
Bra008693
ZmPHD57
Bra020653
Bra011153
AT4G12620
AT4G14700
Bra029471
PtPHD42
OsPHD33
ZmPHD37
ZmPHD56
AT5G09790
ZmPHD1
AT5G24330
Bra009752
OsPHD8
PtPHD23
PtPHD35
OsPHD10
PtPHD54
PtPHD62
AT2G27980
Bra011988
Bra034381
PtPHD20
PtPHD40
AT2G36720
PtPHD30
Bra017226
PtPHD28
AT2G37520
AT3G53680
OsPHD6
OsPHD32
ZmPHD41
PtPHD8
AT5G58610
PtPHD43
OsPHD34
ZmPHD38
OsPHD20
ZmPHD27

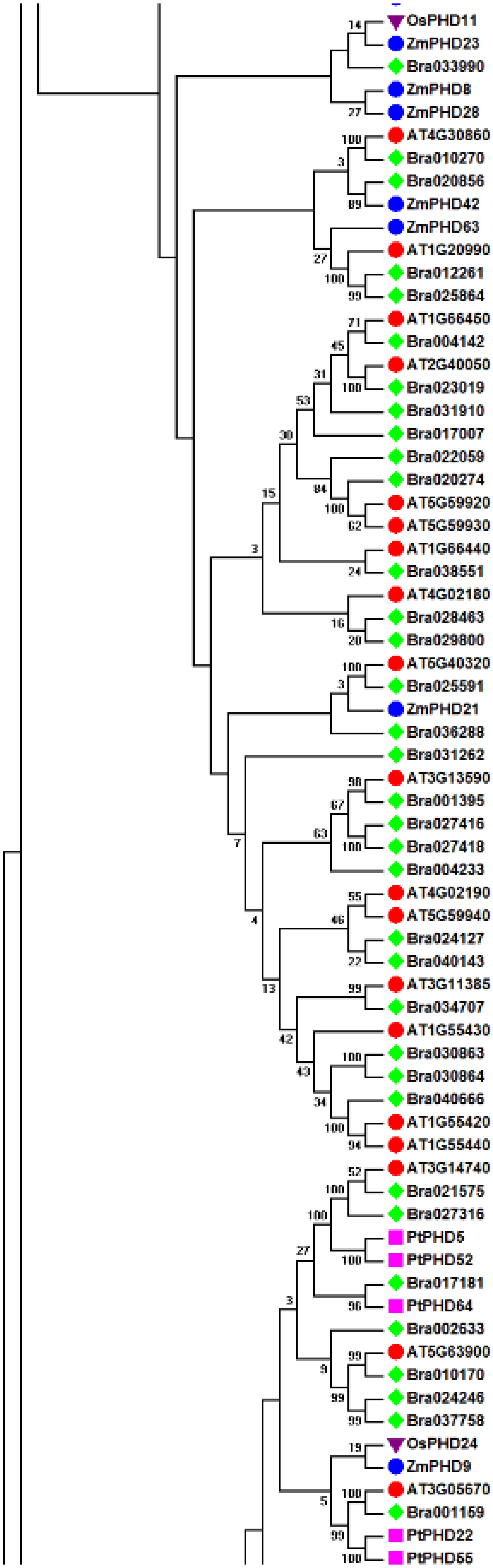

OsPHD11
ZmPHD23
Bra033990
ZmPHD8
ZmPHD28
AT4G30860
Bra010270
Bra020856
ZmPHD42
ZmPHD63
AT1G20990
Bra012261
Bra025864
AT1G66450
Bra004142
AT2G40050
Bra023019
Bra031910
Bra017007
Bra022059
Bra020274
AT5G59920
AT5G59930
AT1G66440
Bra038551
AT4G02180
Bra028463
Bra029800
AT5G40320
Bra025591
ZmPHD21
Bra036288
Bra031262
AT3G13590
Bra001395
Bra027416
Bra027418
Bra004233
AT4G02190
AT5G59940
Bra024127
Bra040143
AT3G11385
Bra034707
AT1G55430
Bra030863
Bra030864
Bra040666
AT1G55420
AT1G55440
AT3G14740
Bra021575
Bra027316
PtPHD5
PtPHD52
Bra017181
PtPHD64
Bra002633
AT5G63900
Bra010170
Bra024246
Bra037758
OsPHD24
ZmPHD9
AT3G05670
Bra001169
PtPHD22
PtPHD55

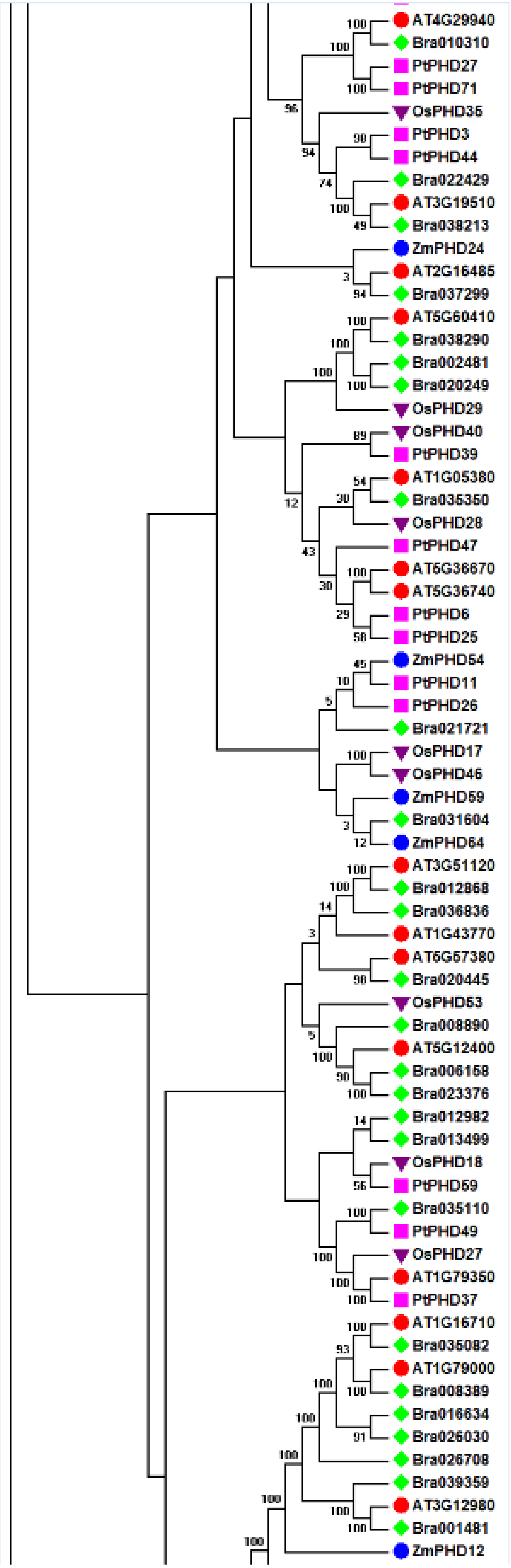
AT4G29940
Bra010310
PtPHD27
PtPHD71
OsPHD35
PtPHD3
PtPHD44
Bra022429
AT3G19510
Bra038213
ZmPHD24
AT2G16485
Bra037299
AT5G60410
Bra038290
Bra002481
Bra020249
OsPHD29
OsPHD40
PtPHD39
AT1G05380
Bra035350
OsPHD28
PtPHD47
AT5G36670
AT5G36740
PtPHD6
PtPHD25
ZmPHD54
PtPHD11
PtPHD26
Bra021721
OsPHD17
OsPHD46
ZmPHD59
Bra031604
ZmPHD64
AT3G51120
Bra012868
Bra036836
AT1G43770
AT5G57380
Bra020445
OsPHD53
Bra008890
AT5G12400
Bra006158
Bra023376
Bra012982
Bra013499
OsPHD18
PtPHD59
Bra035110
PtPHD49
OsPHD27
AT1G79350
PtPHD37
AT1G16710
Bra036082
AT1G79000
Bra008389
Bra016634
Bra026030
Bra026708
Bra039359
AT3G12980
Bra001481
ZmPHD12

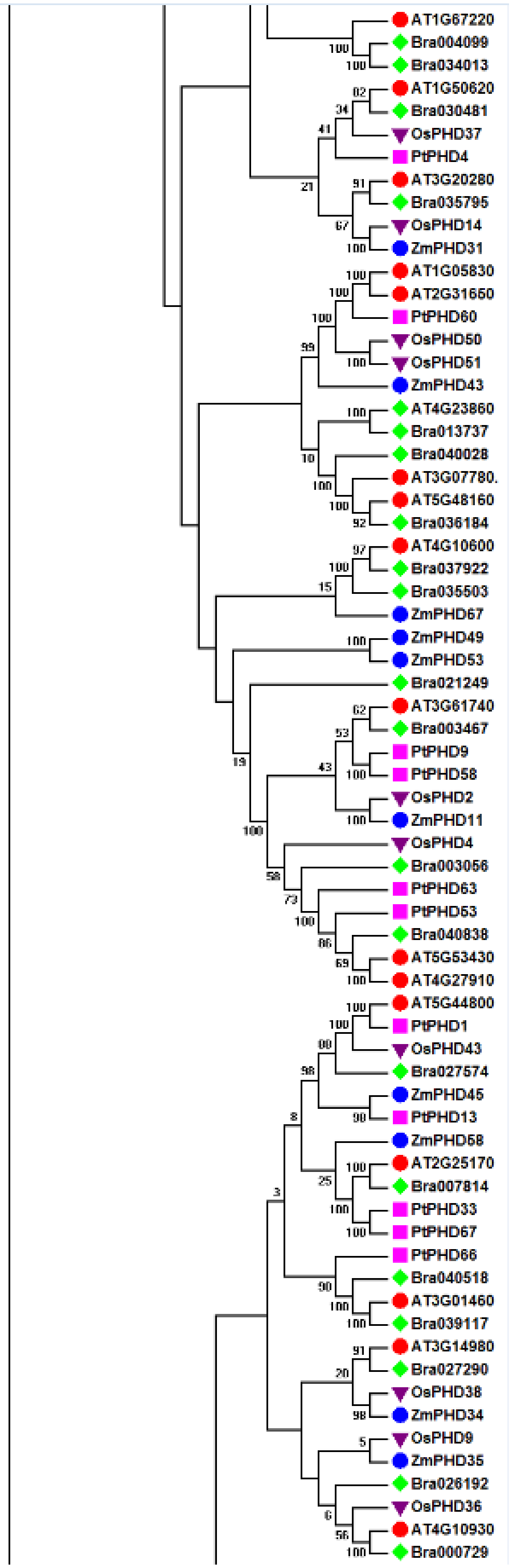
AT1G67220
Bra004099
Bra034013
AT1G50620
Bra030481
OsPHD37
PtPHD4
AT3G20280
Bra035795
OsPHD14
ZmPHD31
AT1G05830
AT2G31660
PtPHD60
OsPHD50
OsPHD51
ZmPHD43
AT4G23860
Bra013737
Bra040028
AT3G07780.
AT5G48160
Bra036184
AT4G10600
Bra037922
Bra035503
ZmPHD67
ZmPHD49
ZmPHD53
Bra021249
AT3G61740
Bra003467
PtPHD9
PtPHD58
OsPHD2
ZmPHD11
OsPHD4
Bra003056
PtPHD63
PtPHD53
Bra040838
AT5G53430
AT4G27910
AT5G44800
PtPHD1
OsPHD43
Bra027574
ZmPHD45
PtPHD13
ZmPHD68
AT2G25170
Bra007814
PtPHD33
PtPHD67
PtPHD66
Bra040518
AT3G01460
Bra039117
AT3G14960
Bra027290
OsPHD38
ZmPHD34
OsPHD9
ZmPHD35
Bra026192
OsPHD36
AT4G10930
Bra000729

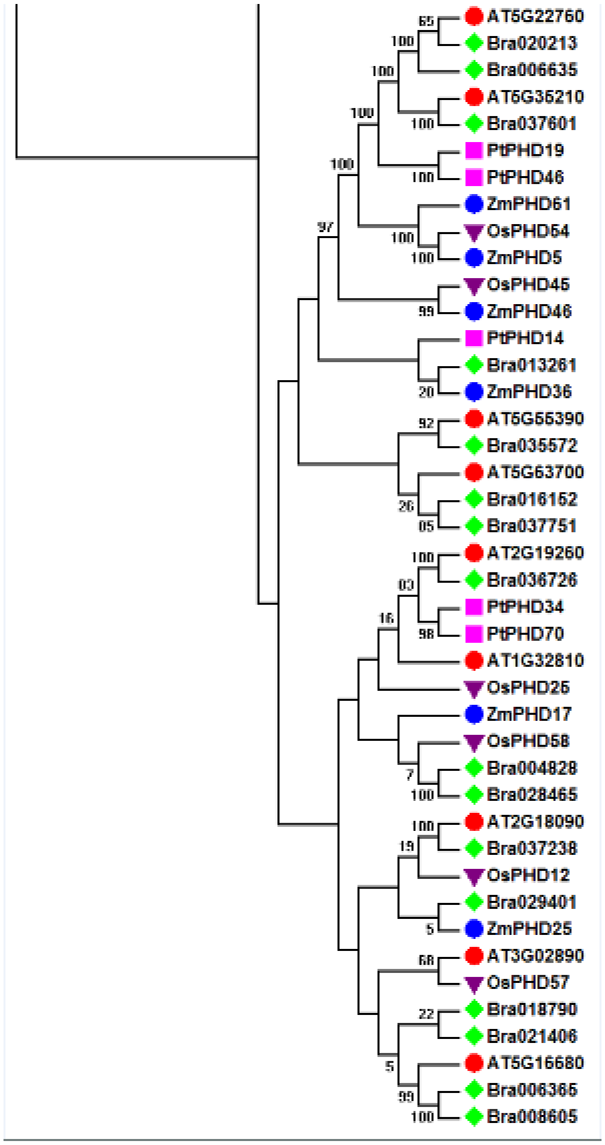
AT5G22760
Bra020213
Bra006635
AT5G35210
Bra037601
PtPHD19
PtPHD46
ZmPHD61
OsPHD54
ZmPHD5
OsPHD45
ZmPHD46
PtPHD14
Bra013261
ZmPHD36
AT5G55390
Bra035572
AT5G63700
Bra016152
Bra037751
AT2G19260
Bra036726
PtPHD34
PtPHD70
AT1G32810
OsPHD25
ZmPHD17
OsPHD58
Bra004828
Bra028465
AT2G18090
Bra037238
OsPHD12
Bra029401
ZmPHD25
AT3G02890
OsPHD57
Bra018790
Bra021406
AT5G16680
Bra006365
Bra008605

Fig. S5 Phylogenetic tree analyses of all 145 *Brassica rapa* PHD finger proteins and a few PHD finger proteins from other species previously characterized as stress or plant development related. See the main text of the paper for corresponding references of the known genes from maize (Zm-), poplar (Pt-), soybean (Gm-), alfalfa (Mt-), *Arabidopsis* (At-) and rice (Os-). * indicates the gene is selected for qRT-PCR analysis under salt and drought stress treatments.

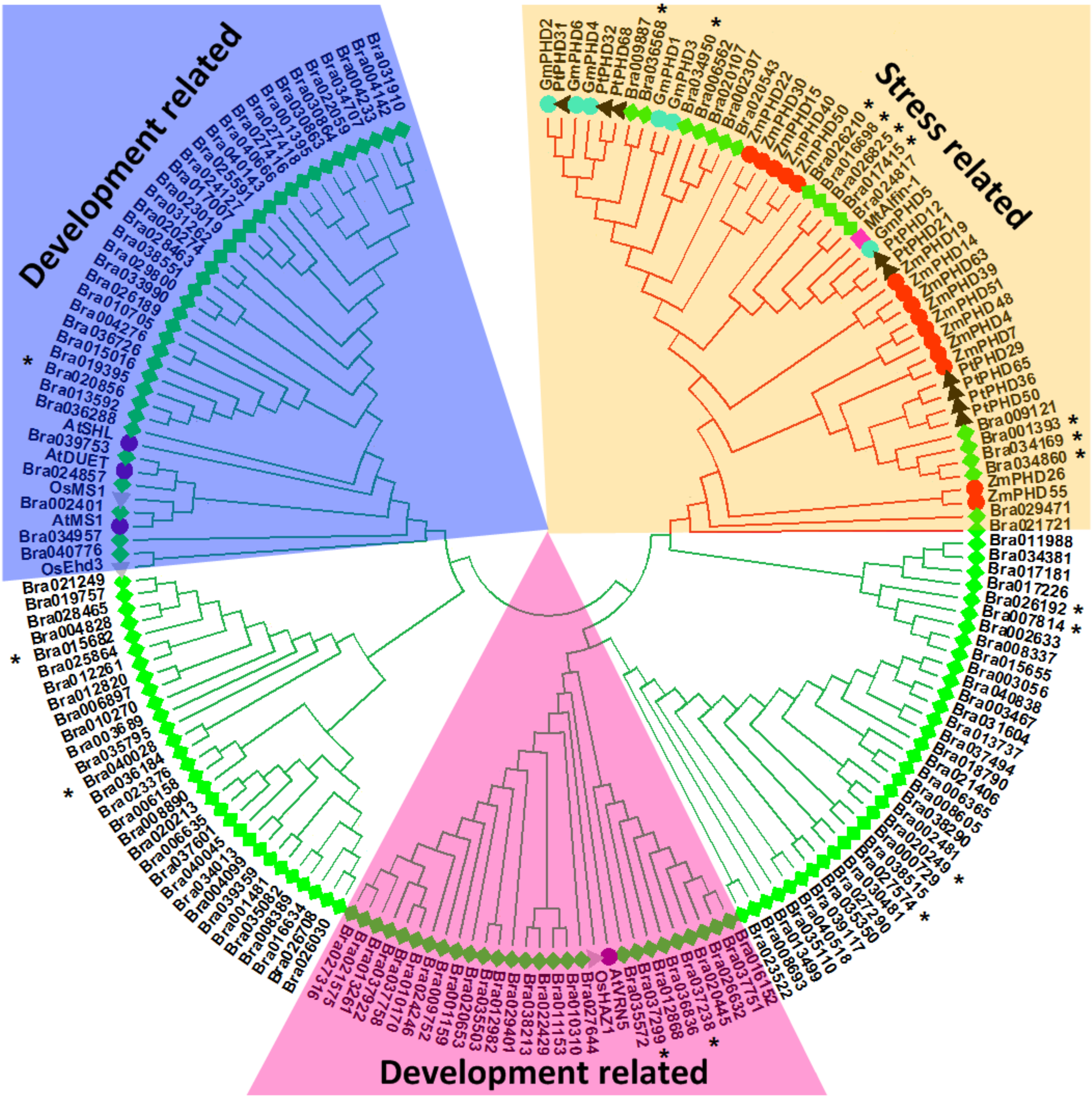

Supplement: Supplementary file 1 — Additional file 1: Fig. S1. Multiple sequence alignment of 233 PHD domains from 145 PHD finger proteins of Brassica rapa. Fig. S2. Sequence logo of the overrepresented motif found in 233 PHD domains of Brassica rapa. Fig. S3. Phylogenetic tree based on multiple sequence alignment of 233 PHD domains from 145 putative PHD finger proteins of Brassica rapa. Fig. S4. Phylogenetic tree based on multiple sequence alignment of PHD finger proteins from Arabidopsis thaliana, Brassica rapa, Oryza sativa, Populus trichocarpa and Zea mays. Fig. S5. Phylogenetic tree analyses of all 145 Brassica rapa PHD finger proteins and a few PHD finger proteins from other species previously characterized as stress or plant development related. [file 12864_2019_6080_MOESM1_ESM.pdf]
